# Genome Complexity Reduction High-Throughput Genome Sequencing of Green Iguana (*Iguana iguana*) Reveal a Paradigm Shift in Understanding Sex-Chromosomal Linkages on Homomorphic X and Y Sex Chromosomes

**DOI:** 10.3389/fgene.2020.556267

**Published:** 2020-10-20

**Authors:** Tassika Koomgun, Nararat Laopichienpong, Worapong Singchat, Thitipong Panthum, Rattanin Phatcharakullawarawat, Ekaphan Kraichak, Siwapech Sillapaprayoon, Syed Farhan Ahmad, Narongrit Muangmai, Surin Peyachoknagul, Prateep Duengkae, Tariq Ezaz, Kornsorn Srikulnath

**Affiliations:** ^1^Laboratory of Animal Cytogenetics and Comparative Genomics, Department of Genetics, Faculty of Science, Kasetsart University, Bangkok, Thailand; ^2^Special Research Unit for Wildlife Genomics, Department of Forest Biology, Faculty of Forestry, Kasetsart University, Bangkok, Thailand; ^3^Mildpets Animal Hospital, Bangkok, Thailand; ^4^Department of Botany, Kasetsart University, Bangkok, Thailand; ^5^Department of Fishery Biology, Faculty of Fisheries, Kasetsart University, Bangkok, Thailand; ^6^Institute for Applied Ecology, University of Canberra, Canberra, ACT, Australia; ^7^Center for Advanced Studies in Tropical Natural Resources, National Research University, Kasetsart University, Bangkok, Thailand; ^8^Center of Excellence on Agricultural Biotechnology, Bangkok, Thailand; ^9^Amphibian Research Center, Hiroshima University, Higashihiroshima, Japan; ^10^Omics Center for Agriculture, Bioresources, Food and Health, Kasetsart University, Bangkok, Thailand

**Keywords:** DArTseq^TM^, Iguanoidea, SNP, super-sex chromosome, non-recombination

## Abstract

The majority of lizards classified in the superfamily Iguanoidea have an XX/XY sex-determination system in which sex-chromosomal linkage shows homology with chicken (*Gallus gallus*) chromosome 15 (GGA15). However, the genomics of sex chromosomes remain largely unexplored owing to the presence of homomorphic sex chromosomes in majority of the species. Recent advances in high-throughput genome complexity reduction sequencing provide an effective approach to the identification of sex-specific loci with both single-nucleotide polymorphisms (SNPs) and restriction fragment presence/absence (PA), and a better understanding of sex chromosome dynamics in Iguanoidea. In this study, we applied Diversity Arrays Technology (DArTseq^TM^) in 29 phenotypic sex assignments (14 males and 15 females) of green iguana (*Iguana iguana*). We confirmed a male heterogametic (XX/XY) sex determination mode in this species, identifying 29 perfectly sex-linked SNP/PA loci and 164 moderately sex-linked SNP/PA loci, providing evidence probably indicative of XY recombination. Three loci from among the perfectly sex-linked SNP/PA loci showed partial homology with several amniote sex chromosomal linkages. The results support the hypothesis of an ancestral super-sex chromosome with overlaps of partial sex-chromosomal linkages. However, only one locus among the moderately sex-linked loci showed homology with GGA15, which suggests that the specific region homologous to GGA15 was located outside the non-recombination region but in close proximity to this region of the sex chromosome in green iguana. Therefore, the location of GGA15 might be further from the putative sex-determination locus in green iguana. This is a paradigm shift in understanding linkages on homomorphic X and Y sex chromosomes. The DArTseq platform provides an easy-to-use strategy for future research on the evolution of sex chromosomes in Iguanoidea, particularly for non-model species with homomorphic or highly cryptic sex chromosomes.

## Introduction

The genetic framework for sex organ differentiation is highly conserved, but a diversity of sex determination is observed in amniotes (O’Meally et al., 2012; Ezaz et al., 2017). The underlying mechanisms can broadly be divided into two main categories: genotypic sex determination and temperature-dependent sex determination. Diverse and rapid evolution of sex determination has, however, been reported even within closely related species (Inamdar et al., 2012; Altmanová et al., 2017). The turnover rate of sex-determination mechanisms differs among lineages (Pokorná and Kratochvíl, 2014; Rovatsos et al., 2014b, 2019; Altmanová et al., 2017; Natri et al., 2019). Squamate reptiles exhibit variability in sex-determination modes, with a rapid turnover of sex-determination mechanisms (Rovatsos et al., 2014b; Schartl et al., 2016; Ezaz et al., 2017; Singchat et al., 2020b). In addition to gecko lizards and skinks, multiple variants of sex chromosome systems have been identified in iguanian lizards, including male heterogamety (XX/XY) in the majority of iguanid lizards and female heterogamety (ZZ/ZW) in many agamid lizards (Ezaz et al., 2009a,b; Gamble, 2010; Alföldi et al., 2011; Rovatsos et al., 2014a; Altmanová et al., 2017; Matsubara et al., 2019; Srikulnath et al., 2019; Singchat et al., 2020b). The study of sex-determination systems and sex chromosomes in iguanian lizards has been difficult because many have homomorphic sex chromosomes that cannot be identified easily using conventional karyotyping (Brandley et al., 2006; Castiglia et al., 2013; Singchat et al., 2020b). The green anole (*Anolis carolinensis*, ACA), is a highly diversified lizard in the superfamily Iguanoidea. The green anole was the first non-avian reptile selected for whole-genome sequencing, which reveals that the species possesses a homomorphic XX/XY sex-chromosome system (Alföldi et al., 2011). The X chromosome of *A. carolinensis* (ACAX) was identified by observation of differences in fluorescent *in situ* hybridization (FISH) signals on microchromosomes between male and female individuals. The known X-linked region is probably homologous to chicken chromosome 15 (*Gallus gallus*, GGA15) (Alföldi et al., 2011). Studies of the partially comparative gene constituents of sex chromosomes between males and females using quantitative polymerase chain reaction (qPCR) analysis have revealed a pattern of different gene dosages in ACAX-linked regions in the majority of iguanoid lizards, which probably exhibit the XX/XY sex-determination system (Alföldi et al., 2011; Gamble et al., 2014; Rovatsos et al., 2014a,b; Altmanová et al., 2017). These results suggest that sex-chromosomal linkage homology is highly conserved in lizards of Iguanoidea.

Genome sequence analyses and cross-species chromosome mapping have, however, detected genomic convergence in which unrelated sex chromosomes share linkage homologies across distantly related lineages. This might involve genomic regions orthologous to squamate chromosome 2 (SR2) and snake W sex chromosomes (Matsubara et al., 2006, 2012, 2019; Srikulnath et al., 2009a,b, 2013, 2014, 2015; Alföldi et al., 2011; Pokorná et al., 2011; Uno et al., 2012; Matsuda et al., 2015; Ezaz et al., 2017; Singchat et al., 2018, 2020a,b). Comparison of genome-wide single-nucleotide polymorphisms (SNPs) between males and females of the Siamese cobra revealed female-specific loci that also show partial homology with a variety of amniote sex chromosomes, including the ACA X-linked region as GGA15 (Laopichienpong et al., 2020), supporting the hypothesis that the Siamese cobra W sex chromosome is derived from a larger ancestral amniote super-sex chromosome. Interestingly, the co-hybridization signals of the same sex-chromosomal linkage homology are also observed for different chromosomes, as seen in cDNA and bacterial artificial chromosome (BAC) chromosomal maps (Matsuda et al., 2005; Matsubara et al., 2006, 2012, 2019; Srikulnath et al., 2009a,b, 2013, 2014, 2015; Uno et al., 2012; Singchat et al., 2018, 2020a,b). Patterns of FISH signals for the same BACs on multiple different chromosome pairs might result from the presence of common genomic elements (O’Meally et al., 2010; Ezaz et al., 2017; Singchat et al., 2018, 2020a,b; Matsubara et al., 2019). These findings suggest the possibility that chromosomal rearrangement, such as fission or translocation, might have occurred in an ancestral amniote super-sex chromosome, which has been evolutionarily conserved after divergence ∼320 million years ago (MYA) (Hedges and Vidal, 2009). This has resulted in a variety of lineage-specific sex-chromosomal linkages among amniotes (O’Meally et al., 2012; Pokorná and Kratochvíl, 2014; Ezaz et al., 2017). It is therefore necessary to understand whether incidences of partial sex-chromosomal linkage observed on the sex chromosome of one lineage are also observed on both autosomes and/or sex chromosomes in a separate lineage, because this might influence molecular sexing based on sex-linked gene development (Singchat et al., 2018, 2020b; Matsubara et al., 2019). Taken together, this raises the questions of whether (a) a small number of partial gene constituents of sex-chromosomal linkage are reliable to prove sex-chromosomal linkage of each lineage based on the supposition of an ancestral super-sex chromosome, and (b) in addition to molecular dosage copy-number estimation and cytogenetic approaches, can comparison of genome sequences or genome-wide SNPs between males and females be used to investigate sex-chromosomal linkage?

For an improved understanding of the relationship of sex-chromosomal linkages and species that exhibit genotypic sex determination with homomorphic sex chromosomes, the green iguana (*Iguana iguana*) was selected as a suitable non-model species to determine sex identification and sex-linked markers (Rovatsos et al., 2014b). The green iguana is a large, arboreal, mostly herbivorous species of lizard in the Iguanoidea, and is found over a broad geographical area in South America (Falcón et al., 2013). Green iguana are sexually dimorphic in maturity, with external morphological traits such as femoral pores, heavy jowls, and a hemipenis bulge at the base of the tail (Kaplan, 1994; Rivas et al., 1996). The diploid chromosome number of the green iguana is 34, comprising six pairs of macrochromosomes and 11 pairs of microchromosomes. No difference in chromosome morphology between males and females is observed (Cohen et al., 1967; Singchat et al., 2020b); however, based on qPCR analyses, the green iguana is an XX/XY species and shares sex-chromosomal linkage across lizards in the Iguanoidea with GGA15, except for members of two genera of basilisks in the family Corytophanidae (Rovatsos et al., 2014a; Nielsen et al., 2019). These findings lead to our prediction that the X and Y sex chromosomes of the green iguana are microchromosomes and highly cryptic (i.e., morphologically poorly differentiated) (Singchat et al., 2020b). In light of this scenario, we tested the hypotheses that (i) the green iguana exhibits an XX/XY sex-chromosome system and harbors male-specific loci, and (ii) sex-chromosomal linkage homology should include a large proportion of the genomic segment GGA15. Recent genotyping-by-sequencing (GBS), using next-generation sequencing technologies such as Diversity Arrays Technology (DArTseq^TM^) developed by Diversity Arrays Technology, Pty Ltd. (Canberra, ACT, Australia), is an effective method with a key genome complexity reduction concept for identification of sex-linked loci in non-model species using SNP loci, to generate restriction site-specific presence/absence (PA) markers. This method is achieved through combining restriction enzymes that separate low copy sequences (most informative for marker discovery and typing) from the repetitive fraction of the genome. The markers generated by DArTseq reveal loci linked to a particular sex, thereby enabling accurate identification of loci tightly linked to the sex-determination region of the sex chromosomes. This is a useful molecular tool for disclosure of sex-determination modes in non-model species with cryptic sex chromosomes such as green iguana (Lambert et al., 2016). Other GBS technology such as RADseq used only one enzyme with different internal technical procedures and still retains more repetitive fractions (Gamble and Zarkower, 2014; Gamble et al., 2015). In this study, we addressed these two hypotheses using DArTseq^TM^. Findings provide novel insights into the evolutionary history of sex chromosomes, representing sex-chromosomal linkage in green iguana in relation to other amniotes. Identification of male-specific loci contributes to our understanding of sex-chromosome evolution.

## Materials and Methods

### Specimens and DNA Extraction

Twenty-nine individuals (14 males and 15 females) of adult green iguana (*I. iguana*) from several clutches were collected from Chonburi Iguana Farm, Thailand (13°25′02.4′′ N, 101°02′32.7′′ E). The sex of each green iguana was identified morphologically and confirmed by mating observation (Falcón et al., 2013). Animal care and all experimental procedures were approved by the Animal Experiment Committee, Kasetsart University, Thailand (approval no. ACKU61-SCI-023) and conducted in accordance with the Regulations on Animal Experiments at Kasetsart University. Blood samples were collected from the ventral tail vein using a 23-gauge needle attached to 3 ml disposable syringes. The syringes contained 10 mM ethylenediaminetetraacetic acid for DNA extraction, and total genomic DNA was extracted following the standard salting-out protocol as described previously (Supikamolseni et al., 2015). Each DNA sample was evaluated by gel electrophoresis for the presence of high-molecular-weight DNA and then stored at −20°C until required for DArTseq^TM^ library construction and qPCR analysis.

### Molecular Sexing by Quantitative Real-Time PCR

Molecular sexing by qPCR was performed on five males and five females green iguanas selected randomly, following the method of Rovatsos et al. (2014b) based on comparison of differences in gene dosage between male and female genomes. Females (XX) have twice as many copies of genes linked to the X-specific portion of sex chromosomes as males (XY), whereas genes in autosomal or pseudoautosomal regions are present in equal copy numbers in both sexes. Four primer pairs for four X-linked loci of *A. carolinensis* (*TMEM132D*, *CCDC92*, *ATP2A2*, and *PEBP1*) were applied to identify sex assignment. The single-copy gene *GAPDH*, which was expected to be located on green iguana chromosome 3, corresponding to chicken chromosome 1, was used as the reference gene (Criscuolo et al., 2009; Singchat et al., 2020b) for normalization of gene dosages in the qPCR analyses (Supplementary Table S1). Each qPCR amplification was performed using 10 μl of 2 × KAPA SYBR^®^ FAST qPCR Master Mix (Kapa Biosystems, Cape Town, South Africa), 0.25 μM primers, and 25 ng genomic DNA. Thermal cycling conditions were 95°C for 3 min, followed by 44 amplification cycles of 95°C for 15 s, 56°C for 30 s, and 72°C for 30 s, ending with a melting curve analysis to monitor for potential non-specific products. The dosage of each studied gene (*R*) was calculated from crossing point values and subsequently normalized to the dosage of the *GAPDH* gene from the same DNA sample, based on the equation: *R* = [2^Cp gene^/2^Cp^ ^GAPDH^]^–1^ (Cawthon, 2002). The relative gene dosage ratio (*r*) between the two sexes for each gene was calculated by dividing the average gene dosage in the male (*R*_male_) by the average dosage in the female (*R*_female_), i.e., *r* = *R*_male_/*R*_female_. A relative gene dosage ratio of 0.5 was expected for X-linked genes degenerated on the Y chromosome and 1.0 for autosomal or pseudoautosomal genes. Statistical differences in copy number between male and female green iguanas were examined using a Wilcoxon signed-rank test with R version 3.4.4 statistical software and the “stats” package (R Core Team, 2019).

Technical control qPCRs were run in triplicate for each primer set to ensure there was no contamination. Standard curves were produced for gene-specific sex primers and *GAPDH* primers using the pooled genomic DNA of 10 randomly selected green iguana individuals to ensure consistent rates of amplification over a wide range of DNA concentrations. Threefold serial dilutions were generated ranging from 25 to 0.00025 ng/μl, with five different concentrations providing a linear dynamic range (Supplementary Figure S1). According to best practice guidelines (Nolan et al., 2006), the reaction was considered in relation to a straight line with *R*^2^ exceeding 0.976 for *TMEM132D*, 0.98 for *CCDC92*, 0.9446 for *ATP2A2*, 0.967 for *PEBP1*, and 0.9879 for *GAPDH*, and fitted to the values obtained. The efficiency (*E*) of sex-specific primer amplification, calculated as *E* = -1 + 10^(–1/slope)^, was 0.9169-2.0762, and the efficiency of *GAPDH* amplification was 1.0004 (Supplementary Table S2). To test intraplate repeatability, the intra-assay coefficient of variation (CV;%) was determined in triplicate for 10 samples. The percentage CV for each sample was calculated by determining the standard deviation (SD) of each technical triplicate *C*_p_ value for *GAPDH* (G) and sex-specific primers (S), then dividing by the triplicate mean and multiplying by 100. The intra-assay CV should be less than 10% (Schultheiss and Stanton, 2009; Hanneman et al., 2011; Singchat et al., 2019). In the present study, the intra-assay CVs were 1.6267–3.6803 and 1.9351% for G and S, respectively (Supplementary Table S2). All samples fell within the concentration range generated by the standard curve. The starting concentrations of S and the reference gene G were used to calculate the relative copy number and, in turn, the S/G ratio was calculated as the copy number of sex-specific primer repeats (Cawthon, 2002; Criscuolo et al., 2009). To test intra- and inter-plate repeatability, samples from 10 green iguana individuals were run in technical triplicate on three different plates in order to monitor plate-to-plate variation. The plate mean for each sample was calculated and subsequently the overall mean, SD, and CV were computed. Inter-assay CVs of less than 15% are generally acceptable (Schultheiss and Stanton, 2009; Hanneman et al., 2011). In the present study, the inter-assay CV (*n* = 3) was 3.43394%, whereas the intra-assay CV was 0.8696–4.2789 and 4.3715% for S and G, respectively (Supplementary Table S3).

### Development of DArTseq^TM^

A detailed description of the DArTseq^TM^ methodology is provided by Jaccoud et al. (2001) and often produces 69 bp sequences. Genotyping of multiple loci was performed using DArTseq^TM^ for SNP loci and *in silico* DArT (presence/absence of restriction fragments in the representation; PA loci) to determine candidate sex-specific loci in male and female individuals. A set of green iguana specimens was used to develop the DArTseq^TM^ arrays with approximately 100 ng genomic DNA from each individual. The DNA samples were subjected to digestion/ligation reactions as described by Kilian et al. (2012) and digested with *Pst*I and a second restriction enzyme (*Sph*I). Ligation reactions were then performed using two adaptors: a *Pst*I-compatible adaptor consisting of an Illumina flow-cell attachment sequence, primer sequence, and a unique barcode sequence, and a *Sph*I-compatible adaptor consisting of an Illumina flow-cell attachment region. Ligated fragments were then PCR amplified using an initial denaturation at 94°C for 1 min, followed by 30 cycles of 94°C for 20 s, 58°C for 30 s, and 72°C for 45 s, with a final extension step at 72°C for 7 min. Equimolar amounts of amplification products from each individual were pooled and subjected to Illumina’s proprietary cBot^1^ bridge PCR, followed by sequencing on an Illumina HiSeq 2000 platform. Single-read sequencing was run for 77 cycles.

Sequences were processed using proprietary DArTseq^TM^ analytical pipelines (Ren et al., 2015). Initially, the HiSeq 2000 output (FASTQ file) was processed to filter poor-quality sequences. Two thresholds of quality were applied. For the barcode region (which allowed parsing of sequences into specific sample libraries), we applied stringent selection (minimum Phred pass score of 30, minimum pass percentage 75). For the remainder of the sequence, relaxed thresholds were applied (minimum Phred pass score 10, minimum pass percentage 50). This calling quality was assured by high average read depth per locus (average across all markers was over 30 reads/locus). These parameters provide the optimal filtering step for potential paralogs as they select markers with Mendelian patterns of behavior. Moreover, the sex-specific region of green iguana is expected to be very cryptic as homomorphic sex chromosomes. The whole read quality with low stringent thresholds must allow for all possible analyses. Approximately 2,000,000 sequences per individual were identified and used in marker calling. Finally, identical sequences were collapsed into “fastqcoll” files that were used in the secondary proprietary pipeline (DArTsoft14) for SNP and PA loci calling; DArTsoft14 implements a “reference-free” algorithm. All unique sequences from the set of fastqcoll files were identified and clustered by sequence similarity at a distance threshold of three base variations using an optimized (fast) clustering algorithm (in many cases more than 1 billion sequences are clustered within minutes). The sequence clusters were then parsed into SNP and *in silico* DArTseq^TM^ markers utilizing a range of metadata parameters derived from the quantity and distribution of each sequence across all samples in the analysis. A high degree of technical replication was included in the DArTseq^TM^ genotyping process, enabling reproducibility scores to be calculated for each candidate marker. Outputs by DArTsoft14 were then filtered on the basis of reproducibility values, average count for each sequence (sequencing depth), balance of average counts for each SNP allele, and call-rate (i.e., proportion of samples for which the marker was scored).

### Marker Selection and DArTseq^TM^ Analysis

Sex-specific loci were derived from analysis of SNP codominant markers and PA as dominant markers. The SNP data were coded as “0” for the reference allele homozygote (the most common allele), “1” for the SNP allele homozygote, “2” for the heterozygote, or “−” as the double null/null allele homozygote (absence of a SNP fragment in the genomic representation). The PA data were coded as “1” for presence, “0” for absence, or “−” for putative heterozygosity. For sex-linked markers in an XX/XY sex-determination system, reference alleles were located on the X-chromosome. In this study, “SNP alleles” were those that showed polymorphism relative to the reference allele. In an XX/XY system, SNP alleles should be those associated with the Y sex chromosome, and located in or near to the male-determination region if the allele is tightly Y-specific. If the two sex chromosomes are recombined, SNP alleles should appear occasionally on the X chromosome. Some males might then be homozygous for SNP alleles at particular loci, with females being heterozygous, exhibiting a copy of the SNP allele. The probability of a female being homozygous for a SNP allele was low. For evaluation of loci associated with an XX/XY system, SNP loci that maximized female homozygosity at the reference allele, maximized male heterozygosity, and minimized homozygosity for the SNP allele in both sexes were expected. Loci for which frequencies of female homozygosity for the reference allele were at least 70% were retained, while those for which homozygosity at the SNP allele was more than 30% with heterozygosity more than 30% were discarded. For males, loci for which frequency of homozygosity for the reference allele was at most 30% and heterozygosity was at least 70% were retained. However, this allowed sex-linked loci to show higher degrees of SNP allele homozygosity for males if recombination occurred. For PA markers, loci that had restriction fragments sequenced in at least 70% of males and not sequenced in at least 70% of females were selected. The SNP and PA loci sequenced for 80, 90, and 100% of males were also included in a separate data set. A filtering criterion of 100% probability was applied to define perfectly sex-linked loci, whereas 70–90% probabilities were deemed to define moderately sex-linked loci. A similar, but opposite, approach was performed to target loci with a ZZ/ZW system.

The Hamming distance was calculated to determine pairwise differences in the number of SNP and PA loci between male and female individuals using the “rdist” function of R version 3.5.1 statistical software (Nolan et al., 2006). Heatmaps were plotted using the “ggplot2” R package (Nolan et al., 2006). The Hamming distance represents the number of pairwise differences between all individuals across all loci. To examine the genetic association between each locus and phenotypic sex assignment from SNP and PA loci, the Cochran–Armitage trend test (CATT) was performed using the “catt” function of R version 3.5.1 implemented in the “HapEstXXR” package (Nolan et al., 2006; Sven and Klaus, 2019). The CATT results were similar to those of a chi-square test to assess whether the proportion of different genotypes followed the null expectation. Polymorphism information content (PIC), as an index for evaluation of the informativeness of SNP and PA loci, was calculated for each locus and ranged from 0 (fixation of one allele) to 0.5 (frequencies of both alleles are equal).

### Estimation of Random Sex-Linkage

To estimate the probability that candidate loci might show random association with sex under a small sample size (Gamble et al., 2015) the formula *P*_i_ = 0.5*^n^* explains the probability of a locus being sex-linked by chance, where *P* is the probability for a given locus, i is sex-linked, 0.5 is the probability that either a male was either homozygous or a female was heterozygous at a given locus, and *n* is the number of individuals sequenced at the locus. After sequencing, we multiplied *P* by the number of high-quality SNPs produced to estimate the number of SNPs expected to exhibit a sex-linked pattern by chance.

### Comparison of Potential Sex-Linked Loci

A Chi-square test was performed on the PA loci and on heterozygosity for SNP loci data to determine whether the four groups (100:0, 90:10, 80:20, and 70:30 of male:female ratios) had associated categorical variable. Differences in mean heterozygosity among the groups were determined using the Kruskal–Wallis test, with the Nemeyi test used for *post hoc* comparisons. All candidate loci were plotted against each individual using the “glPlot” function in the “dartR” R package (Gruber and Georges, 2019; R Core Team, 2019).

### Homology Searching

For all sex-linked loci that attained the filtering criteria and showed a statistically significant association with sex phenotype, a Basic Local Alignment Search Tool (BLAST) search of the National Center for Biotechnology Information (NCBI) database was performed to investigate homologies of sex-linked SNP/PA loci against the green anole lizard (*A. carolinensis*) (AAWZ00000000.2) (Alföldi et al., 2011) and chicken (*G. gallus*) (AADN00000000.5) (International Chicken Genome Sequencing Consortium, 2004) reference genomes. Using the BLASTn program, all loci were then used to search the NCBI database^2^ and RepBase version 19.11 (Bao et al., 2015) (Genetic Information Research Institute^3^), a specialized nucleotide sequence collection for repeated or other significant sequences, which reports only E-values lower than 0.001 and queries coverage with similarity more than 70%.

## Results

### Comparison of Dosages of ACA X-Linked Genes by qPCR

**FIGURE 1:**
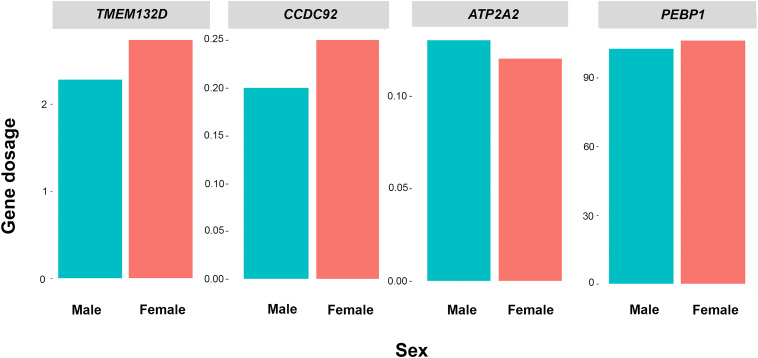
Relative gene dosage ratios between male and female individuals of green iguana (*Iguana iguana*) for *TMEM132D*, *CCDC92*, *ATP2A2*, and *PEBP1*. Blue values indicate males and red values indicate females.

Comparison of the dosages of four genes (*TMEM132D*, *CCDC92*, *ATP2A2*, and *PEBP1*) among five males and five females by qPCR showed that the green iguana exhibited non-sex-specific dosages (Table 1, Figure 1 and Supplementary Tables S4–S7) for ACA X-linked genes, although sex-chromosomal homology was confirmed from the similarity to ACAX and correspondence to GGA15 (Rovatsos et al., 2014b).

**TABLE 1:** Relative gene dosage ratios (*r*) among five males and five females individuals for four genes in green iguana (*Iguana iguana*).

**Primer pair**	**Gene symbol**	**Gene**	**Position in *Anolis carolinensis***	***r***	**Wilcoxon signed-rank test**
TMEM132D_1	*TMEM132D*	Transmembrane protein 132D	X chromosome	0.83	*W* = 8, *p* = 0.7302
CCDC92_1	*CCDC92*	Coiled-coil domain-containing protein 92	X chromosome	1.21	*W* = 8, *p* = 1
ATP2A2_1	*ATP2A2*	Sarcoplasmic/endoplasmic reticulum calcium ATPase 2	X chromosome	0.78	*W* = 7, *p* = 1
PEBP1_1	*PEBP1*	Phosphatidylethanolamine-binding protein 1	X chromosome	0.96	*W* = 16, *p* = 0.5476

### Sex-Determination System and Identification of Sex-Specific Loci in Green Iguana

We sequenced 80,540 SNP loci and 83,292 PA loci. The PIC ranged from 0.49 to 0.50 for both SNP and PA markers. The overall distribution of PIC values was asymmetrical and skewed toward the higher values (Supplementary Figure S2). To determine whether XY or ZW determined the sex of green iguana individuals, we compared a number of SNPs and PAs after filtering with different male:female ratios as criteria. For the ZZ/ZW type of sex determination, filtering using the criterion of 30:70 male:female resulted in identification of 15 SNP loci and 457 PA loci as female-specific. The proportional pairwise Hamming distance between males and females using sex-specific SNP and PA loci (under the null exclusive model) showed within-sex distances of 0.499 ± 0.035 in males and 0.416 ± 0.036 in females for SNP loci, and 0.425 ± 0.033 in males and 0.410 ± 0.036 in females for PA loci. Between-sex distances showed 0.654 ± 0.021 for SNP loci and 0.649 ± 0.022 for PA loci. The CATT verified significant loci association with phenotypic sex for 15 SNP loci (χ^2^ = 5.00–9.00, *p* < 0.001) and 450 PA loci (χ^2^ = 5.00–16.00, *p* < 0.001). Three PA loci were specifically associated with females based on the criterion of 20:80 male:female, whereas no SNP loci were identified as female-specific. The proportional pairwise Hamming distance between males and females using sex-specific SNP and PA loci revealed within-sex distances of 0.341 ± 0.037 in males and 0.248 ± 0.042 in females for PA loci. Between-sex distances were 0.768 ± 0.023 for PA loci. The CATT verified a significant locus association with sex phenotype for three PA loci (χ^2^ = 13.00–16.00, *p* < 0.001). No SNP or PA loci associated with females were identified with the criteria of 10:90 or 0:100 male:female. Chi-square tests indicate that these filtering criteria showed no significant differences in males (χ^2^ = 1.1969, *p* = 0.5497) and females (χ^2^ = 0.83319, *p* = 0.6593) (Table 2 and Supplementary Figure S3).

**TABLE 2:** DArT analysis for 14 males and 15 females individuals of green iguana (*Iguana iguana*) (ZZ/ZW sex-determination type).

	**20 males: 80 females**		**30 males: 70 females**	
	PA^1^	SNPs^2^	PA^1^	SNPs^2^
Total number of DArT analyses	83,292	80,540	83,292	80,540
Sex-specific loci	3	0	457	15
Polymorphic information content	0.49–0.50	–	0.49–0.50	0.49–0.50
Overall mean distance between males and females	0.768 ± 0.023	–	0.649 ± 0.022	0.654 ± 0.021
Overall mean distance within females	0.248 ± 0.042	–	0.410 ± 0.036	0.416 ± 0.036
Overall mean distance within males	0.341 ± 0.037	–	0.425 ± 0.033	0.499 ± 0.035

*^1^Presence–absence data. ^2^Single nucleotide polymorphism data.*

**FIGURE 2:**
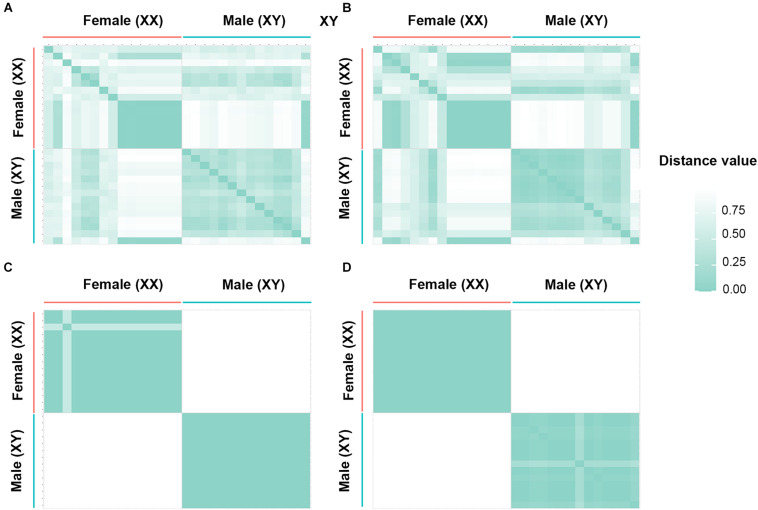
Hamming distance between male and female individuals of green iguana (*Iguana iguana*). **(A)** Single-nucleotide polymorphism (SNP) loci filtered by the criterion of 70:30 (males:females), **(B)** restriction fragment presence/absence (PA) loci filtered by the criterion of 70:30 (males:females), **(C)** SNP loci filtered by the criterion of 0:100 (males:females), and **(D)** PA loci filtered by the criterion of 0:100 (males:females).

By contrast, for the XX/XY type of sex determination, filtering using the criterion 70:30 male:female yielded 74 SNP loci and 908 PA loci as male-specific. The proportional pairwise Hamming distance between male and female green iguanas using sex-specific SNP and PA loci revealed within-sex distances of 0.429 ± 0.020 in males and 0.531 ± 0.030 in females for SNP loci, and 0.357 ± 0.025 in males and 0.423 ± 0.030 in females for PA loci. Between-sex distances were 0.654 ± 0.021 for SNP loci and 0.649 ± 0.022 for PA loci. The CATT verified a significant locus association with sex phenotype for 59 SNP loci (χ^2^ = 5.00–29.00, *p* < 0.001) and 902 PA loci (χ^2^ = 5.00–30.00, *p* < 0.001). Filtering with the criterion of 80:20 male:female yielded four SNP loci and 43 PA loci as male-specific. The Hamming distance between males and females using sex-specific SNP and PA loci revealed within-sex distances of 0.135 ± 0.019 in males and 0.357 ± 0.026 in females for SNP loci, and 0.135 ± 0.010 in males and 0.070 ± 0.007 in females for PA loci. Between-sex distances were 0.882 ± 0.013 for SNP loci and 0.940 ± 0.005 for PA loci. The CATT verified a significant locus association with sex phenotype for four SNP loci (χ^2^ = 7.00–30.00, *p* < 0.001) and 43 PA loci (χ^2^ = 13.00–30.00, *p* < 0.001). Filtering with the criterion 90:10 male:female identified two SNP loci and 33 PA loci as male-specific. The Hamming distance between males and females using sex-specific SNP and PA loci revealed within-sex distances of 0.000 ± 0.000 in males and 0.067 ± 0.017 in females for SNP loci, and 0.085 ± 0.008 in males and 0.000 ± 0.000 in females for PA loci. Between-sex distances were 1.000 ± 0.000 for SNP loci and 0.987 ± 0.003 for PA loci. The CATT verified a significant locus association with sex phenotype for two SNP loci (χ^2^ = 28.00–29.00, *p* < 0.001) and 33 PA loci (χ^2^ = 24.00–30.00, *p* < 0.001). Two SNP loci and 27 PA loci were associated with males based on the criterion of 100:0 male:female. The proportional pairwise Hamming distance between male and female green iguana using sex-specific SNP and PA loci revealed lower within-sex distances (0.000 ± 0.000 in males and 0.067 ± 0.017 in females for SNP loci, and 0.000 ± 0.000 in males and 0.058 ± 0.008 in females for PA loci). By contrast, higher between-sex distances of 1.000 ± 0.000 for both SNP and PA loci were observed. The CATT verified a significant locus association with sex phenotype for two SNP loci (χ^2^ = 28.00–29.00, *p* < 0.001) and 27 PA loci (χ^2^ = 27.00–30.00, *p* < 0.001) (Table 3, Figure 2 and Supplementary Figure S3).

**TABLE 3:** DArT analysis for 14 males and 15 females individuals of green iguana (*Iguana iguana*) (XX/XY sex-determination type).

	**70 males: 30 females**		**80 males: 20 females**		**90 males: 10 females**		**100 males: 0 females**	
	PA^1^	SNPs^2^	PA^1^	SNPs^2^	PA^1^	SNPs^2^	PA^1^	SNPs^2^
Total number of DArT analyses	83,292	80,540	83,292	80,540	83,292	80,540	83,292	80,540
Sex-specific loci	902	59	43	4	33	2	27	2
Polymorphic information content	0.49–0.50	0.49–0.50	0.49–0.50	0.49–0.50	0.49–0.50	0.49–0.50	0.49–0.50	0.49–0.50
Overall mean distance between males and females	0.673 ± 0.018	0.712 ± 0.015	0.940 ± 0.005	0.882 ± 0.013	0.987 ± 0.003	1.000 ± 0.000	1.000 ± 0.000	1.000 ± 0.000
Overall mean distance within females	0.423 ± 0.030	0.531 ± 0.030	0.070 ± 0.007	0.357 ± 0.026	0 ± 0	0.067 ± 0.017	0.000 ± 0.000	0.067 ± 0.017
Overall mean distance within males	0.357 ± 0.025	0.429 ± 0.020	0.135 ± 0.010	0.135 ± 0.019	0.085 ± 0.008	0.000 ± 0.000	0.058 ± 0.008	0.000 ± 0.000

*^1^Presence–absence data. ^2^Single nucleotide polymorphism data.*

For the PA loci data, Chi-square tests showed that the filtering criteria yielded no significant differences in males (χ^2^ = 0.19, *p* = 0.98) and females (χ^2^ = 0.62, *p* = 0.89). However, for the SNP loci data, the filtering criteria yielded significantly different percentages of heterozygosity in both males and females (Kruskal–Wallis test, *p* ≤ 0.001). The 70:30 filtering criterion produced significantly lower heterozygosity than the other filtering criteria in males (Nemeyi test, *p* ≤ 0.001). In females, the 70:30 filtering criterion resulted in significantly lower heterozygosity than the other filtering criteria (*p* = 0.008), except for the 80:20 criterion (*p* = 0.45). A glPlot revealed that the sample group showed greater similarity between sexes when perfectly and moderately sex-linked loci were considered (Supplementary Figure S4).

### Estimation of Random Sex-Linkage

Across a range of sample sizes and loci, 29 individuals were required to minimize the probability of selecting less than one spurious sex-linked marker. The probability *P*_i_ that a single locus exhibited a sex-linked pattern by chance was 1.86 × 10^–9^ from 173,144 loci (including SNP and PA loci), whereas the expected sex-linkage was estimated to be 3.23 × 10^–4^. Random sex-linked markers in green iguana were, therefore, more frequent than the expected values in this study.

### Homology of Putative Sex-Specific Loci

Based on perfectly sex-linked loci, male sex-specific loci of green iguana showed sequence homology with the reference genomes of green anole and chicken, on the basis of global BLAST analyses of NCBI databases (accessed 22 March, 2020). We observed that six of 29 male-specific SNP/PA loci corresponded to GGA5, GGA18, GGA27, GGA28, and GGAZ (Supplementary Table S8), whereas three out of 29 SNP/PA loci were homologous with the putative candidate genes *LIN52* (E-values 8 × 10^–05^ and similarity 72%), *OGFOD3* (E-values 5.9 and similarity 23%), and *RAPGEFL1* (E-values 5 × 10^–07^ and similarity 76%). In addition, four out of 29 SNP/PA loci were homologous to transposable elements consisting of one type of long terminal repeat (LTR) retrotransposons and two types of non-LTR retrotransposons (Supplementary Table S9).

Based on moderately sex-linked loci, we found 164 of 932 loci from SNP/PA male-specific loci corresponding to GGA1, GGA2, GGA3, GGA4, GGA5, GGA6, GGA7, GGA8, GGA9, GGA11, GGA12, GGA15, GGA17, GGA18, GGA20, GGA22, GGA26, GGA27, GGA33, and GGAZ (Supplementary Table S10), whereas 59 SNP/PA loci were homologous with putative functional genes. Moreover, 12 SNP loci and 126 PA loci were homologous to transposable elements consisting of four types of DNA transposons, one type of endogenous retroviruses, four types of LTR retrotransposons, and seven types of non-LTR retrotransposons (Supplementary Table S9).

## Discussion

Evolution of sex chromosomes of lizards in Iguanoidea indicates linkage homology similar to ACAX, which is homologous to GGA15 (Alföldi et al., 2011; Rovatsos et al., 2014a,b; Ezaz et al., 2017). The majority of these sex chromosomes evolved before the divergence of iguanian lizards ∼73–93 MYA (Townsend et al., 2011; Zheng and Wiens, 2016), and after the divergence of Acrodonta and Pleurodonta ∼123–168 MYA given that Acrodonta possess different sex-determination systems (XX/XY and ZZ/ZW sex chromosomes) (Townsend et al., 2011; Rovatsos et al., 2014b, 2015; Zheng and Wiens, 2016; Srikulnath et al., 2019). It would be advantageous to investigate the function of sequences or genes located in the genomic block of GGA15 because it may contain the same sex-determination gene. Substantial efforts have been devoted to identification of the mechanisms of sex determination and sex chromosome evolution in lizards of the Iguanoidea (Alföldi et al., 2011; Gamble et al., 2014; Rovatsos et al., 2014a,b; Altmanová et al., 2017; Singchat et al., 2020b). Recent evidence based on comparison of partial gene content using a qPCR approach has shown that sex chromosomes of green iguana are highly conserved with many lizards of Iguanoidea (Rovatsos et al., 2014b). The results of qPCR molecular sexing conducted with five males and five females of green iguanas, following the method of Rovatsos et al. (2014b), reveal the copy number of non-sex-specific gene doses in specimens from the same species (*I. iguana*) used in the previous study (Rovatsos et al., 2014b). The efficiency of our qPCR system was also examined, and the results were under the standard baseline for qPCR prediction (Rutledge and Côté, 2003). This finding leads to the prediction that the green iguana in the two studies showed different linkages of sex-chromosome systems based on turnover from another autosomal pair. However, this scenario is difficult to envisage because the results of chromosome painting do not support sex-chromosome turnover in lizards of Iguanoidea (Lisachov et al., 2020). An additional possibility might be an intraspecific polymorphism in sex-determination systems, as in the case of the Japanese wrinkled frog *Glandirana rugosa* (Ogata et al., 2018). DArTseq^TM^ technology is, therefore, an alternative approach as a paradigm shift for examining sex-determination systems and the genetic constitution of sex chromosomes. The high number of SNP/PA loci allowed us to predict sex-specific loci for the green iguana using a sample size of 29 individuals (14 males and 15 females). The probability that one locus would spuriously show sex linkage was 1.86 × 10^–9^. We sampled 3.23 × 10^–4^ sex-specific loci. Although this is likely to represent relatively small sample sizes, identification of any sex-linked loci by chance in the present samples was highly unlikely. Several cases found in the North American green frog (*Rana clamitans*) identified sex-linked loci using small sample sizes (Lambert et al., 2016). To our best knowledge, ours is the first report to demonstrate genome-wide SNP loci in relation to phenotypic sex assignment in green iguana. The X and Y sex chromosomes of green iguana probably differ in genetic composition from each other.

### Confirmation of XY Sex Determination and Characterization of Male-Specific Sex Loci for Green Iguana

The present study identified 193 of 961 SNP/PA loci to be male-specific. Of these loci, 29 were perfectly linked with male determination (100:0, male:female) but not with female determination (0:100, male:female). Thus, across each identified locus all known sex individuals were present in all males and absent in all females. This finding confirms that green iguana exhibit an XX/XY sex-determination system (Rovatsos et al., 2014b). The perfectly sex-linked loci suggest the presence of a distinct male-specific region of the Y chromosome. The majority formed a haplotype that became fixed by the cooperative selection of favorable sex-linked chromosomes with hitchhiking effects on the Y sex chromosome and a focal variant (Ezaz et al., 2017). Chromosomes that carry a certain suite of sexually antagonistic genes are more likely to be recruited as sex chromosomes (Bachtrog et al., 2011). The acquisition of male-specific functions would set the stage for adaptive evolution involving sex-determination regions, and introduce strong selective constraints against the degeneration of Y-linked genes. Moreover, this region would represent the putative non-recombination region of sex chromosomes (Charlesworth, 2017). A BLAST search of the NCBI databases for homologous sequences detected three SNP/PA loci that encoded amino acids (*LIN52*, *OGFOD3*, and *RAPGEFL1*) in the tentative Y-specific fragments. However, there is no direct evidence that these genes are involved in squamate reptile sex determination or sex development. Given the relatively short sequence length of all loci, more detailed research is required to characterize fully these genes and their functionality in green iguana. Fragments of four SNP/PA loci were similar to transposable elements and showed 3.4 to 10.34% homology (Supplementary Table S8 and Supplementary Figure S3). The presence of transposable elements in non-recombination regions might regulate the gene expression pattern of the Y sex chromosome through interruption of the gene structure, resulting in gradual silencing and degeneration of the chromosome (Śliwińska et al., 2016).

The highly conserved karyotype of Iguanoid lizards, with homomorphic sex chromosomes frequently appearing on microchromosomes, suggests that the small proportional size of the putative non-recombination region of the X and Y chromosomes of green iguana might reflect the evolutionarily young status of the sex chromosomes, rather than turnover events with autosomes (Rovatsos et al., 2014b). At least a partially degenerate Y chromosome represents the ancestral situation for recently evolved iguanas. Kruskal–Wallis and Nemenyi tests reveal that a number of perfectly and moderately sex-linked loci were significantly different, especially under filtering criteria of 70:30 and 100:0 (male:female), suggesting that partial recombination of XY sex chromosomes clearly differed from the non-recombination region. Interestingly, sex-chromosome recombination is phenotypically, rather than genotypically, moderated in a male heterogametic species (Perrin, 2009; Sopniewski et al., 2019). In the present study, we observed that the 70:30 filtering criterion yielded significantly lower heterozygosity than the other filtering criteria in males, whereas in females heterozygosity with the 70:30 criterion was no different from that with the 80:20 filtering criterion, which indicates that the rate of recombination region is higher in females than males in green iguana. This suggests that sex-chromosome recombination is suppressed by ‘maleness’ rather than different sex chromosomes (Rodrigues et al., 2018; Sopniewski et al., 2019). A total of 932 out of 173,144 SNP/PA loci were, with regard to sex phenotype, completely discordant with the population of 14 males and 15 females individuals. This finding suggests that linkage might result in ambiguous genetic sexes for certain individuals, and 17.6% of SNP/PA loci showed homology with genes encoding amino acids. Preservation of such genes may be driven by selection in males to maintain two copies of dosage-sensitive genes, as observed in human X and Y chromosomes (Wilson Sayres and Makova, 2013; Wilson et al., 2020). A total of 14.8% of SNP/PA loci were homologous to transposable elements (Supplementary Table S4 and Supplementary Figure S3). The majority of loci involved Gypsy transposable elements, which are often distributed on sex chromosomes in medaka (*Oryzias latipes*), platyfish (*Xiphophorus maculatus*), guppy (*Poecilia reticulata*), Siamese cobra (*Naja kaouthia*), and tilapia (Herpin et al., 2010; Böhne et al., 2012; Chalopin et al., 2015; Laopichienpong et al., submitted data), whereas large numbers of short interspersed nuclear elements were detected on the Y sex chromosomes of frog (Hill et al., 2000; Luo et al., 2017).

### Possibility of an Amniote Super-Sex Chromosome

**FIGURE 3:**
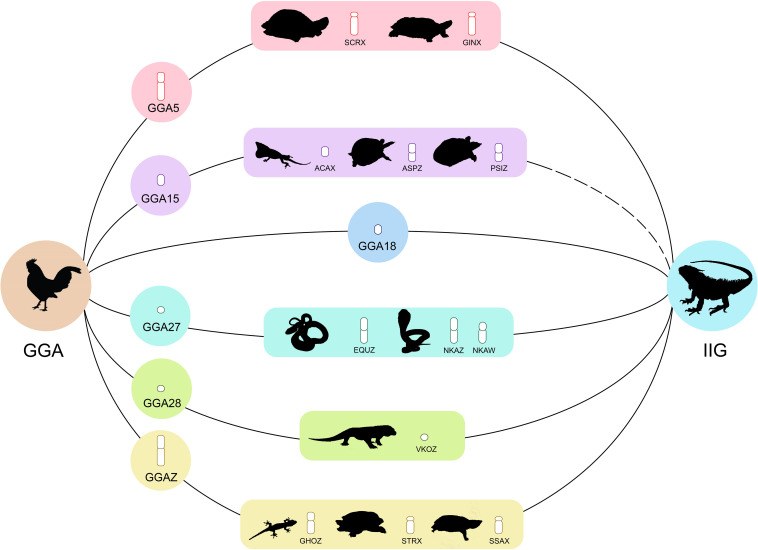
Male-specific loci of green iguana (*Iguana iguana*) showing homologies with chicken (*Gallus gallus*, GGA) and other amniote sex chromosomes. Sex chromosomes of other amniotes shown are Komodo dragon (*Varanus komodoensis*, VKO), Japanese four-striped rat snake (*Elaphe quadrivirgata*, EQU), Monocled cobra (*Naja kaouthia*, NKA), Chinese softshell turtle (*Pelodiscus sinensis*, PSI), giant musk turtle (*Staurotypus salvinii*, SSA), Mexican musk turtle (*Staurotypus triporcatus*, STR), Wood turtle (*Glyptemys insculpta*, GIN), Spiny softshell turtle (*Apalone spinifera*, ASP), and Hokou gecko (*Gekko hokouenesis*, GHO).

Using comparative gene mapping (i.e., chromosome mapping by means of a cytogenetic technique) and whole-genome sequencing, genomic convergence has been detected in which unrelated sex chromosomes share syntenies across distantly related taxa (Voss et al., 2001; Matsuda et al., 2005; Matsubara et al., 2006, 2012, 2019; Srikulnath et al., 2009a,b, 2013, 2014, 2015; Alföldi et al., 2011; Pokorná et al., 2011; Ezaz et al., 2013, 2017; Young et al., 2013; Gamble et al., 2017; Augstenová et al., 2018; Singchat et al., 2018, 2020a,b; Iannucci et al., 2019; Lind et al., 2019; Ahmad et al., 2020). The ACAX-specific region contains genomic content orthologous to genes on GGA15 (Alföldi et al., 2011), which is homologous to the Z chromosome of the Chinese softshell turtle (*Pelodiscus sinensis*) and the spiny softshell turtle (*Apalone spinifera*) (Kawagoshi et al., 2009, 2014). This result led to the prediction of a hypothetical larger ancestral amniote super-sex chromosome with overlaps of partial linkage homology among sex chromosomes of amniotes, whereby multiple translocations have then resulted in the appearance of various sex chromosomes in amniotes (Ezaz et al., 2017; Singchat et al., 2018, 2020a,b; Matsubara et al., 2019; Srikulnath et al., 2019; Ahmad et al., 2020). In the present study, six out of 29 SNP/PA loci that were perfectly sex-linked with phenotypic sex assignment showed partial homology with a number of amniote sex chromosomes (GGA5, GGA18, GGA27, GGA28, and GGAZ) (International Chicken Genome Sequencing Consortium, 2004) (Supplementary Figure S4). The micro-Z chromosomes of *Varanus komodoensis* correspond to GGA28 and the majority of snake Z chromosomes correspond to GGA2 and GGA27 (Pokorná and Kratochvíl, 2014; Singchat et al., 2018). The X chromosomes of the marsh turtle (*Siebenrockiella crassicollis*) and wood turtle (*Glyptemys insculpta*) correspond to GGA5 (Montiel et al., 2017; Singchat et al., 2018). Highly conserved linkage homology of the chicken Z chromosome has been observed for the X chromosomes of the Mexican giant musk turtle (*Staurotypus triporcatus*), the giant musk turtle (*Staurotypus salvinii*), and the Z chromosome of the Hokou gecko (*Gekko hokouenesis*) (Kawai et al., 2009; Kawagoshi et al., 2014; Srikulnath et al., 2015). These results suggest that male-specific loci of the Y sex chromosome in green iguana also share sex-chromosomal linkage homologies with unrelated sex-chromosome-linked regions of other amniotes. Comparative chromosome maps between squamate reptiles and birds using cross-species BAC FISH mapping revealed that GGA5 (CH261-161B22, BAC clone) and GGAZ (CH261-129A16, BAC clone) are mapped on different pairs of green iguana microchromosomes, whereas GGA5 (CH261-161B22, BAC clone) is homologous to the long arm of the W sex chromosome of *N. kaouthia*, *Notechis scutatus* and *Daboia russelii*, and chromosome 1 of *Varanus salvator macromaculatus*, and corresponds to squamate chromosome 2 (SR2) (Srikulnath et al., 2013; Singchat et al., 2018, 2020a,b). These results thus strongly support the hypothesis of an ancestral amniote super-sex chromosome that might have involved genomic regions orthologous to SR2 and the snake W sex chromosome (Ezaz et al., 2017; Singchat et al., 2018, 2020a,b; Matsubara et al., 2019; Ahmad et al., 2020). By contrast, we did not detect any evidence of loci in the non-recombination region homologous to GGA15; however, only one of 961 SNP/PA loci retained with the filtering criterion 90:10 male:female as moderately sex-linked loci was homologous to GGA15. This finding suggests that the specific region containing genetic constituents homologous to GGA15 tends to be located outside the non-recombination region but in close proximity to the non-recombination region of the sex chromosomes in green iguana. The location of GGA15 might therefore be more distant from the putative sex-determination locus in green iguana, differing from the previous hypothesis (Lambert et al., 2016; Rodrigues et al., 2018; Sopniewski et al., 2019; Ahmad et al., 2020). This becomes a paradigm shift in understanding sex-chromosomal linkages on homomorphic X and Y sex chromosomes. Remarkably, data from the Genomicus database (Nguyen et al., 2018^4^) indicate that several genes from GGA15 are localized to autosomes in ACA (e.g., ACACB on ACA1 or SFI1 on ACA3) (Supplementary Figure S5). This might result from multiple translocations (insertions and/or fusions) that appeared during the 250 million years of divergence between ACA and GGA (Rovatsos et al., 2014a) (Figure 3), and might also have occurred in lizards of Iguanoidea. These hypotheses should be tested by observing the behavior of the sex chromosomes of green iguana during male meiosis with cDNA/BAC FISH mapping. Alternatively, partial linkage homology of green iguana Y sex chromosomes to several amniote sex chromosomes might represent random homologies, given that only small sets of genomic regions in a restricted set of species are involved. However, the main uncertainty in this scenario concerns the actual set of sex-chromosomal linkage homologies of green iguana. Additional evidence, such as whole-genome sequencing or molecular combing, is required to identify this linkage homology.

### How Many Functional Gene Markers Are Required for Sex Identification or Sex Chromosomal Linkage Investigation, If the Ancestral Super-Sex Chromosome Hypothesis Is Supported?

Molecular sexing is an attractive approach in evolutionary and ecological research as well as in conservation and breeding for accurate sex identification of adults in monomorphic or slightly sexually dimorphic species. It is also the only method capable of identifying the sex of embryos and juveniles before the development of primary and secondary sexual characteristics (Morinha et al., 2012). Sex chromosomes are conserved in birds, and the majority of mammals and snakes; therefore, established molecular sexing techniques are often applied throughout these groups (Fridolfsson and Ellegren, 1999; Fernando and Melnick, 2001; Morin et al., 2005; Brubaker et al., 2011; Morinha et al., 2012, 2015; Laopichienpong et al., 2017a,b; Tawichasri et al., 2017). However, no such widely applicable technique was thought possible for other amniotes because they show variability in sex-determination systems and/or homomorphic sex chromosomes (Ezaz et al., 2009b; Pokorná and Kratochvíl, 2009; Gamble, 2010; Sarre et al., 2011; Gamble et al., 2015). Comparative genomics have revealed that the ancestral X chromosome is conserved in lizards of Iguanoidea (Gamble et al., 2014; Rovatsos et al., 2014b; Altmanová et al., 2017). These studies suggest a common origin from the same ancestral pair of sex chromosomes, as evidenced by the sex-chromosomal linkage homology block composed of ACAX-specific genes, which has been detected in the sex chromosomes of various lineages (Alam et al., 2018). As mentioned above, the qPCR analysis in the present study was performed on the same species as those included in the study of Rovatsos et al. (2014b). No statistical differences in gene copy number were observed between males and females of green iguana. Ancestral X chromosomes of certain lizards in Iguanoidea underwent inversion (Gamble et al., 2014). It is possible that multiple independent diversification events in the analyzed region among green iguana individuals in the present study (i.e., intraspecific polymorphism) were associated with inversion of the ancestral sex-determination locus or translocation of the analyzed green iguana X-specific genes to the recombination region, as found in the one locus corresponding to GGA15. An additional possibility is the hypothesis of an ancestral super-sex chromosome as revealed by several segments involving sex-chromosomal linkages in several amniote species (Ezaz et al., 2017; Singchat et al., 2018, 2020a,b; Matsubara et al., 2019; Ahmad et al., 2020). The results of genome-wide SNP analysis, as is possible using DArTseq^TM^ technology, provide a large number of sex-specific SNP/PA loci at perfectly and moderately sex-linked loci for identification of sex determination and sex-linked markers, although the method can only be applied to a small group of closely related species (Sopniewski et al., 2019). The use of a small number of genes to identify sex determination and sex-chromosomal linkages might result in errors because many homologous loci across amniote sex chromosomes were observed at Y-specific loci in the current study, while false positive qPCR results might often be obtained in different laboratories (Rovatsos et al., 2014b). Developmental molecular identification and analysis of sex-linked genetic markers based on a PCR approach are required to establish a genotyping tool for practical sexing of individuals in a commercial breeding program and allow accurate determination of sex in populations through insight into the evolutionary history of the Y chromosome (or the W chromosome in a female heterogametic species, such as occurs in snakes) (Laopichienpong et al., 2017a,b; Tawichasri et al., 2017).

## Conclusion

We have successfully identified sex-specific loci on the Y sex chromosome of green iguana using DArTseq^TM^ technology. We identified a notable number of perfectly sex-linked loci given the small portion of the genome. The results confirm the presence of XX/XY sex determination in green iguana. Several male-specific SNP/PA loci of green iguana had partial homology with a number of amniote sex chromosomes (GGA5, GGA18, GGA27, GGA28, and GGAZ), supporting the hypothesis of an ancestral amniote super-sex chromosome. However, this did not concur with the previous prediction hypothesized for a large proportion of the genomic segment GGA15. This represents a paradigm shift in the understanding of sex-chromosomal linkages on homomorphic X and Y sex chromosomes. It remains unclear whether the location of large genomic regions between X-specific and Y-specific fragments is associated with differentiation of sex chromosomes and sex-determination regions. It seems likely that these loci are sex-linked in other populations. A study population with a relatively even ratio of sexes or wild populations is thus required to minimize the probability of false-positives, although the number of samples needed may also depend on the amount of polymorphism present in the population, and will probably vary across species. Practically, a PCR-based approach should be developed from perfectly sex-linked SNP/PA loci as identified in this study, although failure of the PCR validation step has often been observed after DArTseq^TM^ or RADseq bioinformatics analysis (Gamble et al., 2015). Chromosome mapping of sex-specific loci on mitotic and meiotic chromosomes using FISH is required to determine their positions on the sex chromosomes of green iguana. In addition, a high-quality complete genome assembly for green iguana is required to elucidate further the sex-determination mechanism. This approach will provide a solid basis for explaining the sex-determination mechanism and identifying potential sex-determination regions in iguana and other squamate reptiles.

## Data Availability Statement

The full dataset and metadata from this publication are available from the Dryad Digital Repository. Dataset, https://doi.org/10.5061/dryad.j0zpc86b5.

## Ethics Statement

The animal study was reviewed and approved by Animal care and all experimental procedures were approved by the Animal Experiment Committee, Kasetsart University, Thailand (approval no. ACKU61-SCI-023) and conducted in accordance with the Regulations on Animal Experiments at Kasetsart University.

## Author Contributions

TK and KS drafted the manuscript and carried out the lab work. TK, NL, EK, and KS conceived the ideas and designed the methodology and participated in data analysis. All authors reviewed the data and the manuscript and gave final approval for publication.

## Conflict of Interest

The authors declare that the research was conducted in the absence of any commercial or financial relationships that could be construed as a potential conflict of interest.

## Footnotes

1 http://www.illumina.com/products/cbot.html

2 http://blast.ncbi.nlm.nih.gov/Blast.cgi

3 http://www.girinst.org/

4 https://www.genomicus.biologie.ens.fr/genomicus-98.01/cgi-bin/search.pl

## Supplementary Material

Supplementary Figure 1 Standard curves generated for **(A)** *TMEM132D*, **(B)** *CCDC92*, **(C)** *ATP2A2*, **(D)** *PEBP1*, and **(E)** *GAPDH*. Linear dynamic range from 25 to 0.00025 ng/μl genomic DNA concentration extracted from green iguana (*Iguana iguana*) (*n* = 10). Regression lines were calculated as *R*^2^ = 0.9446–0.9879, *p* < 0.001 for *TMEM132D*, *CCDC92*, *ATP2A2*, *PEBP1*, and *GAPDH*.

Supplementary Figure 2 Density PIC plot for **(A)** single-nucleotide polymorphism (SNP) and **(B)** restriction fragment presence/absence (PA) loci.

Supplementary Figure 3 Number of loci in different hypotheses of sex-determination systems after filtering using different male:female ratios as criteria. The *x*-axis indicates the number of loci and the *y*-axis indicates the filtering criteria. **(A)** Restriction fragment presence/absence (PA) loci and **(B)** single-nucleotide polymorphism loci.

Supplementary Figure 4 An index of the two perfectly sex-aligned sequences and the additional 59 sex-related sequences created with the ‘glPlot’ function implemented in the “dartR” R package. Blue indicates homozygosity to the reference allele, purple is indicative of heterozygosity, and red indicates homozygosity to the alternate SNP-containing allele. Sequences two (right of the figure) show perfectly sex-linked sequences; females are indicated in blue and males are indicated in purple.

Supplementary Figure 5 Segmental homology of green anole (*Anolis carolinensis*) and chicken (*Gallus gallus*). Segmental linkage homologies are derived from the Genomicus database (Louis et al., 2014). Arrow indicate chromosome 15 of chicken and autosome of anolis.

Supplementary Table 1 Primers used for the measurement of relative gene dosage by quantitative PCR analysis.

Supplementary Table 2 Inter- and intra-assay coefficient of variation (CV) and efficiency of five genes for dilution standard curve.

Supplementary Table 3 Inter- and intra-assay coefficient of variation (CV) and efficiency for five genes.

Supplementary Table 4 Relative gene dosage ratios between five males and five females individuals of green iguana (*Iguana iguana*) for *TMEM132D*.

Supplementary Table 5 Relative gene dosage ratios between five males and five females individuals of green iguana (*Iguana iguana*) for *CCDC92*.

Supplementary Table 6 Relative gene dosage ratios between five males and five females individuals of green iguana (*Iguana iguana*) for *ATP2A2*.

Supplementary Table 7 Relative gene dosage ratios between five males and five females individuals of green iguana (*Iguana iguana*) for *PEBP1*.

Supplementary Table 8 Gene function and pathway for single-nucleotide polymorphism (SNP) and restriction fragment presence/absence (PA) loci of green iguana (*Iguana iguana*) from a BLAST search of the green anole (*Anolis carolinensis*) genome (100:0, male:female).

Supplementary Table 9 Repeat searches for single-nucleotide polymorphism (SNP) and restriction fragment presence/absence (PA) loci of green iguana (*Iguana iguana*).

Supplementary Table 10 Gene function and pathway for single-nucleotide polymorphism (SNP) and restriction fragment presence/absence (PA) loci of green iguana (*Iguana iguana*) from a BLAST search of the green anole (*Anolis carolinensis*) genome (70:30, male:female).
