# Supplementary material for: Genome Complexity Reduction High-Throughput Genome Sequencing of Green Iguana (Iguana iguana) Reveal a Paradigm Shift in Understanding Sex-Chromosomal Linkages on Homomorphic X and Y Sex Chromosomes
Source: Front Genet. 2020 Oct 20;11:556267. doi: 10.3389/fgene.2020.556267 (PMC7606854; doi:10.3389/fgene.2020.556267)
Supplement: Supplementary Figure 1 — Standard curves generated for (A) TMEM132D, (B) CCDC92, (C) ATP2A2, (D) PEBP1, and (E) GAPDH. Linear dynamic range from 25 to 0.00025 ng/μl genomic DNA concentration extracted from green iguana (Iguana iguana) (n = 10). Regression lines were calculated as R2 = 0.9446–0.9879, p < 0.001 for TMEM132D, CCDC92, ATP2A2, PEBP1, and GAPDH. [file Data_Sheet_1.PDF]

Supplementary Table S1 | Primers used for the measurement of relative gene dosage by quantitative PCR analysis.

Gene name	Gene symbol	Forward primer (5′ – 3′)	Reverse primer (5′ – 3′)	Amplicon size (bp)	Reference
Transmembrane protein 132D	*TMEM132D*	TATCCGAGCAGACCCAAAGTCC	AAGGAGACCCAACTCAGCCAC	183	Rovatsos et al. (2014)
Coiled-coil domain-containing protein 92	*CCDC92*	TAGTCATTCCGCCCATTCCC	GACCACTTTGCTCCCATTCAC	179	Rovatsos et al. (2015)
Sarcoplasmic/endoplasmic reticulum calcium ATPase 2	*ATP2A2*	CAAAGCAGCGGGCATTTAGG	ATCACTGGGGACAACAAGGG	160	Rovatsos et al. (2016)
Phosphatidylethanolamine-binding protein 1	*PEBP1*	GACAGGGCTCCATCGCTAC	CATAGTCATCCCACTCCGCC	188	Rovatsos et al. (2017)
Glyceraldehyde-3-phosphate dehydrogenase	*GAPDH*	AACCAGCCAAGTACGATGACAT	CCATCAGCAGCAGCCTTCA	50	Criscuolo et al. (2009)

Supplementary Table S2 | Inter- and intra-assay coefficient of variation (CV) and efficiency of five genes for dilution standard curve.

Gene	Inter-assay CV (%)[1]	Intra-assay CV (%)[2]	Efficiency[3]
TMEM132D	2.5848	3.6387	0.9169
CCDC92	3.6803	7.5571	0.9353
ATP2A2	2.508	3.0862	2.0762
PEBP	1.6267	1.1476	1.0590
GAPDH	1.9351	1.5869	1.0004

[1]Inter-assay CVs of less than 15 are generally acceptable (Hanneman et al., 2011; Schultheiss and Stanton, 2009).

[2]Intra-assay CVs should be less than 10 (Hanneman et al., 2011; Schultheiss and Stanton, 2009).

[3]Efficiency should be 0.9–1.1 (Rutledge and Côté, 2003).

Supplementary Table S3 | Inter and intra-assay coefficient of variation (CV) for five genes.

Gene	Inter-assay CV (%)[1]	Intra-assay CV (%)[2]
TMEM132D	6.0437	0.8696
CCDC92	1.9859	2.7683
ATP2A2	3.0467	4.2789
PEBP	3.0467	2.3709
GAPDH	3.0467	4.3715

[1]Interassay CVs of less than 15 are generally acceptable (Hanneman et al., 2011; Schultheiss and Stanton, 2009).

[2]Intra-assay CVs should be less than 10 (Hanneman et al., 2011; Schultheiss and Stanton, 2009).

Supplementary Table S4 | Relative gene dosage ratios between five male and five female individuals of green iguana (*Iguana iguana*) for *TMEM132D*.

Sex/ratio	Female1	Female2	Female3	Female4	Female5
Male1	0.2441959	7.675E-05	0.0007132	0.0001023	-
Male2	38594.593	12.130403	112.72523	16.160999	-
Male3	45.921922	0.0144334	0.1341265	0.0192292	-
Male4	964.66813	0.3031983	2.8175563	0.4039426	-
Male5	74.428217	0.023393	0.2173864	0.0311659	-

Supplementary Table S5 | Relative gene dosage ratios between five male and five female individuals of green iguana (*Iguana iguana*) for *CCDC92*.

Sex/ratio	Female1	Female2	Female3	Female4	Female5
Male1	-	-	-	-	-
Male2	2.016239	0.0770058	2.3977258	0.2057392	-
Male3	1.2958412	0.0494918	1.5410235	0.1322291	-
Male4	5.6721233	0.2166343	6.7453294	0.5787897	-
Male5	2.5737803	0.0982999	3.0607578	0.2626314	-

Supplementary Table S6 | Relative gene dosage ratios between five male and five female individuals of green iguana (*Iguana iguana*) for *ATP2A2*.

Sex/ratio	Female1	Female2	Female3	Female4	Female5
Male1	0.033121	-	0.8734047	0.0598848	0.3507058
Male2	-	-	-	-	-
Male3	0.1099561	-	2.8995587	0.1988075	1.1642852
Male4	0.866537	-	22.850703	1.566753	9.1754428
Male5	0.1063334	-	2.8040265	0.1922574	1.1259253

Supplementary Table S7 | Relative gene dosage ratios between five male and five female individuals of green iguana (*Iguana iguana*) for *PEBP1*.

Sex/ratio	Female1	Female2	Female3	Female4	Female5
Male1	0.0011573	5.542E-06	5.739E-05	6.241E-06	3.026E-05
Male2	28.475843	0.136366	0.0587202	0.1535675	0.744552
Male3	1.1841804	0.0056708	0.0587202	0.0063862	0.0309625
Male4	392.68324	1.8804932	19.472056	2.1177026	0.4086362
Male5	15.62854	0.0748424	0.7749753	0.0842832	0.4086362

Supplementary Table S8 | Gene function and pathway for single-nucleotide polymorphism (SNP) and restriction fragment presence/absence (PA) loci of green iguana (*Iguana iguana*) from a BLAST search of the green anole (*Anolis carolinensis*) genome (100:0, male:female).

Locus id	Gene[1,2]	Product	Function	Component	Reference
SNP100064278	*LIN52*	lin-52 DREAM MuvB core complex component	DNA-templated	DRM complex	Tschöp et al. (2011)
PA100029306	*OGFOD3*	2-oxoglutarate and iron dependent oxygenase domain containing 3	L-ascorbic acid binding	integral component of membrane	Liu et al. (2018)
PA100031009	*RAPGEFL1*	Rap guanine nucleotide exchange factor like 1	guanyl-nucleotide exchange factor activity	membrane	Chen et al. (2015)

[1]All genes show sequence similarity with partial sequence of DArT loci more than 23% from database

[2]All genes show an E-value with partial sequence of DArT loci lower than 0.001 from database

Chen, Y., Yin, D., Li., L., Deng, Y. C., and Tian, W. (2015). Screening aberrant methylation profile in esophageal squamous cell carcinoma for Kazakhs in Xinjiang area of China. *Mol*. *Biol*. *Rep*. 42, 457–64. doi: 10.1007/s11033-014-3788-z

Liu, X., Salokas, K., Tamene, F., Jiu, Y., Weldatsadik, R. G., Öhman, T., et al. (2018). An *AP-MS*- and *BioID*-compatible *MAC*-tag enables comprehensive mapping of protein interactions and subcellular localizations. *Nat*. *Commun*. 9, 1188. doi: 10.1038/s41467-018-03523-2

Tschöp, K., Conery, A. R., Litovchick, L., Decaprio, J. A., Settleman, J., Harlow, E., et al. (2011). A kinase shRNA screen links *LATS2* and the *pRB* tumor suppressor. *Genes Dev*. 25,814–30. doi: 10.1101/gad.2000211

Supplementary Table S9 | Repeat searched for single-nucleotide polymorphism (SNP) and restriction fragment presence/absence (PA) loci of green iguana (*Iguana iguana*).

Repeat class	Type	Moderately sex-linked loci; male:female		Perfectly sex-linked loci; male:female		Total
		SNP loci[1]	PA loci[2]	SNP loci[1]	PA loci[2]	
Caulimoviridae	unknown[a]	1	2	-	-	3
DNA transposon	EnSpm/CACTA	-	1	-	-	1
	Mariner/Tc1	-	3	-	-	3
	MuDR	-	2	-	-	2
	hAT	-	4	-	-	4
	unknown[a]	1	-	-	-	1
Endogenous Retrovirus	ERV1	-	1	-	-	1
LTR Retrotransposon	BEL	1	-	-	-	1
	Copia	-	4	-	-	4
	DIRS	-	3	-	-	3
	Gypsy	8	76	1	1	86
Non-LTR Retrotransposon	CR1	1	4	-	1	6
	L1	-	1	1	1	3
	L2	1	8	-	-	9
	Penelope	-	1	-	-	1
	Jockey	-	2	-	-	2
	RTE	-	1	-	-	1
	SINE	-	6	-	-	6

[1] single-nucleotide polymorphism loci

[2] presence-absence

Supplementary Table 10 | Gene function and pathway for single-nucleotide polymorphism (SNP) and restriction fragment presence/absence (PA) loci of green iguana (*Iguana iguana*) from a BLAST search of the green anole (*Anolis carolinensis*) genome (70:30, male:female).

Locus id	Gene[1,2]	Product	Function	Component	Reference
SNP100052186	*CAMSAP1*	calmodulin regulated spectrin associated protein 1	microtubule minus-end binding	colocalizes_with microtubule minus-end	Gaudet et al. (2011)
SNP100068808	*FAM76B*	family with sequence similarity 76 member B	protein binding	nuclear speck	Salichs et al. (2009); Zheng et al. (2016)
SNP100075635	*PCMT1*	protein-L-isoaspartate (D-aspartate) O-methyltransferase	protein-L-isoaspartate (D-aspartate) O-methyltransferase activity	cytoplasm	Gaudet et al. (2011)
SNP100081470	*DNAH9*	dynein axonemal heavy chain 9	ATP-dependent microtubule motor activity, minus-end-directed	axoneme	Gaudet et al. (2011)
SNP100088743	*NUP214*	nucleoporin 214	nuclear export signal receptor activity	nuclear pore	Farjot et al. (1999)
SNP100091437	*KHK*	ketohexokinase	ATP binding	cytoplasm	Le et al. (2016)
SNP100092607	*CCDC88A*	coiled-coil domain containing 88A	G-protein gamma-subunit binding	COPI-coated Golgi to ER transport vesicle	Nguyen et al. (2019)
SNP100095810	*MYO3B*	myosin IIIB	ATP binding	cytoplasm	Dosé and Burnside (2002).
SNP100130983	*HNF4A*	hepatocyte nuclear factor 4 alpha	DNA-binding transcription factor activity	cytoplasm	Hakoda et al. (2003)
PA100000126	*CDK17*	cyclin dependent kinase 17	protein binding	cytoplasm	Chaput et al. (2016)
PA100000162	*CDK17*	cyclin-dependent kinase 17	ATP binding	cytoplasm	Chaput et al. (2016)
PA100003473	*GRB7*	growth factor receptor bound protein 7	RNA binding	cell projection	Chu et al. (2019)
PA100004870	*PLEKHA6*	pleckstrin homology domain containing A6	Unknown	Unknown	–
PA100009224	*SBK2*	serine/threonine-protein kinase SBK2	ATP binding	cytoplasm	Greenman et al. (2007)
PA100012710	*C11ORF52*	chromosome unknown open reading frame	Unknown	extracellular exosome	Tomkins et al. (2018)
PA100020489	*DSE*	dermatan sulfate epimerase	chondroitin-glucuronate 5-epimerase activity	Golgi apparatus	Nakao et al. (2000)

PA100029376	*UBE3B*	ubiquitin protein ligase E3B	ubiquitin conjugating enzyme activity	Unknown	Yilmaz et al. (2018)
PA100029735	*C2H6ORF47*	chromosome 2 C6orf47 homolog	Unknown	Unknown	–
PA100029885	*GCGR*	glucagon receptor	G protein-coupled peptide receptor activity	endosome	Zhang et al. (2018)
PA100029972	*DSCAM*	DS cell adhesion molecule	cell-cell adhesion mediator activity	axon	Allach et al. (2019)
PA100030068	*FGFBP3*	fibroblast growth factor binding protein 3	fibroblast growth factor binding	collagen-containing extracellular matrix	Zhang et al. (2008)
PA100030195	*GREM2*	gremlin 2, DAN family BMP antagonist	BMP binding	extracellular region	
PA100030530	*MRS2*	MRS2, magnesium transporter	magnesium ion transmembrane transporter activity	integral component of membrane	Yamanaka et al. (2016)
PA100030594	*SLC48A1*	solute carrier family 48 member 1	heme binding	endosome membrane	Fogarty et al. (2014)
PA100030945	*PHKB*	phosphorylase kinase regulatory subunit beta	calmodulin binding	cytosol	Wang et al. (2017)
PA100032665	*INSC*	inscuteable homolog (Drosophila)	protein binding	colocalizes_with cell cortex	Katoh et al. (2003)
PA100053534	*FAM131B*	family with sequence similarity 131 member B	protein binding	cytosol	Nagase et al. (1998)
PA100060894	*IRF8*	interferon regulatory factor 8	DNA-binding transcription factor activity, RNA polymerase II-specific	autophagy	Ye et al. (2018)
PA100065669	*NAIP*	NLR family, apoptosis inhibitory protein (naip)	ATP binding	basolateral plasma membrane	Kano et al. (2018)
PA100067962	*INSL5*	insulin like 5 (insl5)x	G protein-coupled receptor binding	cellular_component	Bicer et al. (2019)
PA100070558	*SEMA5A*	semaphorin 5A	chondroitin sulfate proteoglycan binding	extracellular exosome	
PA100074787	*RECQL5*	RecQ like helicase 5	ATP binding	RNA polymerase II, holoenzyme	Newman et al. (2017)
PA100078094	*STARD13*	StAR related lipid transfer domain containing 13	GTPase activator activity	cytosol	Wolosz et al. (2019)
PA100079991	*CCDC80*	coiled-coil domain containing 80	fibronectin binding	basement membrane	Liu et al. (2018)
PA100082252	*NRP1*	neuropilin 1 (nrp1)	GTPase activator activity	axon	Huang et al. (2019)

PA100083147	*CDK17*	cyclin dependent kinase 17	protein binding	cytoplasm	Chaput et al. (2016)
PA100087301	*PSTK*	phosphoseryl-tRNA kinase	ATP binding	Unknown	Carlson et al. (2004)
PA100091076	*DOCK7*	dedicator of cytokinesis 7	Rac GTPase binding	colocalizes_with COP9 signalosome	Perrault et al. (2014)
PA100095755	*GPS1*	G protein pathway suppressor 1	GTPase inhibitor activity	COP9 signalosome	Dubois et al. (2016)
PA100097679	*ENKUR*	enkurin, TRPC channel interacting protein	SH3 domain binding	-	Tomkins et al. (2018)
PA100098005	*HCFC1*	host cell factor C1	RNA polymerase II distal enhancer sequence-specific DNA binding	colocalizes_with Ada2/Gcn5/Ada3 transcription activator complex	Kapuria et al. (2018)
PA100098450	*FGGY*	FGGY carbohydrate kinase domain containing	D-ribulokinase activity	cell	Cai et al. (2014)
PA100099234	*PTK2*	protein tyrosine kinase 2	ATP binding	apical plasma membrane	Diaz et al. (2019)
PA100100127	*PTPRE*	protein tyrosine phosphatase, receptor type E	protein binding	cytoplasm	Bhattarai et al. (2017)
PA100101514	*RASGRP1*	RAS guanyl releasing protein 1	Ras guanyl-nucleotide exchange factor activity	Golgi apparatus	Somekh et al. (2018)
PA100118003	*PDZD2*	PDZ domain containing 2	Unknown	cell-cell junction	Leung et al. (2009)
PA100118779	*LAMA1*	laminin subunit alpha 1	extracellular matrix structural constituent	basement membrane	Puchalapalli et al. (2019)
PA100119501	*OSGEPL1*	O-sialoglycoprotein endopeptidase like 1	N(6)-L-threonylcarbamoyladenine synthase activity	EKC/KEOPS complex	Liao et al. (2014)
PA100119518	*LSAMP*	limbic system-associated membrane protein	protein binding	anchored component of membrane	Petrovics et al. (2015)
PA100121090	*CDK17*	cyclin-dependent kinase 17	ATP binding	cytoplasm	Chaput et al. (2016)
PA100124305	*CDK17*	cyclin-dependent kinase 17	ATP binding	cytoplasm	Chaput et al. (2016)
PA100125648	*SLC5A7*	solute carrier family 5 member 7	choline binding	axon	Jones et al. (2018)
PA100131387	*ZNF366*	zinc finger protein 366	DNA binding	nucleus	Ansems et al. (2012)
PA100136133	*SLC4A1*	solute carrier family 4 member 1 (Diego blood group)	anion transmembrane transporter activity	Z disc	Kalli et al. (2008)

PA100138888	*EPS15*	epidermal growth factor receptor pathway substrate 15	SH3 domain binding	AP-2 adaptor complex	Dai et al. 2015
PA100139447	*NOXRED1*	NADP-dependent oxidoreductase domain containing 1	molecular_function	cellular_component	Chen et al. (2019)
PA100146780	*Il7R*	interleukin 7 receptor (il7r)	antigen binding	clathrin-coated vesicle membrane	Fu et al. (2019)
PA100146957	*MYLK*	myosin light chain kinase	ATP binding	actin cytoskeleton	Wallace et al. (2019)
PA100147430	*ACTN2*	actinin alpha 2	FATZ binding	Z disc	Fan et al. (2019)

[1]All genes show sequence similarity with partial sequence of DArT loci more than 70% from database
[2]All genes show an E-value with partial sequence of DArT loci lower than 0.001 from database

Allach, E., Khattabi, L., Backer, S., Pinard, A., Tsatsaris, V., Vaiman, D., et al. (2019). A genome-wide search for new imprinted genes in the human placenta identifies *DSCAM* as the first imprinted gene on chromosome 21. *Eur*. *J*. *Hum*. *Genet*. 27,49–60 doi: 10.1038/s41431-018-0267-3

Bhattarai, N., McLinden, J. H., Xiang, J., Mathahs, M. M., Schmidt, W. N., Kaufman, T. M., et al. (2017). Hepatitis C virus infection inhibits a Src-kinase regulatory phosphatase and reduces T cell activation in vivo. *PLoS Pathog*. 13, 1006232. doi: 10.1371/journal.ppat.1006232

Bicer, M., Alan, M., Alarslan, P., Guler, A., Kocabas, G. U., Imamoglu, C., et al. (2019). Circulating insulin-like peptide 5 levels and its association with metabolic and hormonal parameters in women with polycystic ovary syndrome. *J*. *Endocrinol*. *Invest*. 42, 303–312 doi: 10.1007/s40618-018-0917-x

Carlson, B. A., Xu, X. M., Kryukov, G. V., Rao, M., Berry, M. J., Gladyshev, V. N., et al. (2004). Identification and characterization of phosphoseryl-tRNA [*Ser*]Sec kinase. *Proc*. *Natl*. *Acad*. *Sci*. *USA*. 101, 12848–12853. doi: 10.1073/pnas.0402636101

Cai, B., Tang, L., Zhang, N., and Fan, D. (2014). Single-nucleotide polymorphism rs6690993 in *FGGY* is not associated with amyotrophic lateral sclerosisin a large Chinese cohort. *Neurobiol*. *Aging*. 35, 1512.e3–1512.e4. doi: 10.1016/j.neurobiolaging.2013.12.018

Chaput, D., Kirouac, L., Stevens, S. M. Jr., and Padmanabhan, J. (2016). Potential role of *PCTAIRE-2*, *PCTAIRE-3* and *P-Histone H4* in amyloid precursor protein-dependent Alzheimer pathology. *Oncotarget* 7, 8481–8497. doi:10.18632/oncotarget.7380

Chen, K., Liu, H., Liu, Z., Bloomer, W., Amos, C., Lee, J. E., et al. (2019). Genetic variants in glutamine metabolic pathway genes predict cutaneous melanoma-specific survival. *Mol*. *Carcinog*. 58, 2091-2103. doi: 10.1002/mc.23100

Chu, P. Y., Tai, Y. L., and Shen, T. L. (2019). *Grb7*, a Critical mediator of *EGFR/ErbB* signaling, in cancer development and as a potential therapeutic target. *Cells* 8, 435. doi: 10.3390/cells8050435

Dai, X., Liu, Z., and Zhang, S. (2015). Over-expression of *EPS15* is a favorable prognostic factor in breast cancer. *Mol*. *Biosyst*. 11, 2978–2985. doi:10.18632/oncotarget.14776

Diaz Osterman, C. J., Ozmadenci, D., Kleinschmidt, E. G., Taylor, K. N., Barrie, A. M., Jiang, S., et al. (2019). *FAK* activity sustains intrinsic and acquired ovarian cancer resistance to platinum chemotherapy. *Elife*. 8, e47327. doi: 10.7554/eLife.47327

Dosé, A. C., and Burnside, B. (2002). A class III myosin expressed in the retina is a potential candidate for Bardet-Biedl syndrome. *Genomics* 79, 621–624. doi: 10.1006/geno.2002.6749

Dubois, E. L., Gerber, S., Kisselev, A., Harel-Bellan, A., and Groisman, R. (2016). UV-dependent phosphorylation of *COP9*/signalosome in UV-induced apoptosis. *Oncol*. *Rep*. 35, 3101–3105. doi: 10.3892/or.2016.4671

Fan, L. L., Huang, H., Jin, J.Y., Li, J. J., Chen, Y. Q., and Xiang, R. (2019). Whole-exomesSequencing identifies a novel mutation (*p.L320R*) of alpha-actinin 2 in a chinese family with dilated cardiomyopathy and ventricular tachycardia. *Cytogenet*. *Genome Res.* 157, 148–152. doi: 10.1159/000496077

Farjot, G., Sergeant, A., and Mikaélian, I. (1999). A new nucleoporin-like protein interacts with both *HIV-1* Rev nuclear export signal and *CRM-1*. *J Biol*. *Chem*. 274, 17309–17317. doi: 10.1074/jbc.274.24.17309

Fogarty, F. M., O'Keeffe, J., Zhadanov, A., Papkovsky, D., Ayllon, V., and O'Connor, R. (2014). *HRG-1* enhances cancer cell invasive potential and couples glucose metabolism to cytosolic/extracellular pH gradient regulation by the vacuolar-H(+) ATPase. *Oncogene* 33, 4653–4663. doi: 10.1038/onc.2013.403

Fu, G. M., Chen, D. D., Wu, C., Wang, M., Pan, Z. X., Peng, X. Y., et al. (2019). Mutation and clinical feature of *IL-7R* in adult patients with acute lymphoblastic leukemia. *Zhongguo*. *Shi*. *Yan*. *Xue*. *Ye*. *Xue*. *Za*. *Zhi*. 27, 1416–1423. doi: 10.19746/j.cnki.issn.1009-2137.2019.05.010

Gaudet, P., Livstone, M. S., Lewis, S. E., and Thomas, P D. (2011). Phylogenetic-based propagation of functional annotations within the Gene Ontology consortium. *Brief*. *Bioinform*. 12, 449–462 doi: 10.1093/bib/bbr042

Greenman, C., Stephens, P., Smith, R., Dalgliesh, G. L., Hunter, C., Bignell, G., et al. (2007). Patterns of somatic mutation in human cancer genomes. *Nature* 446, 153–158. doi: 10.1038/nature05610

Hakoda, T., Yamamoto, K., Terada, R., Okano, N., Shimada, N., Suzuki, T., et al. (2003) A crucial role of hepatocyte nuclear factor-4 expression in the differentiation of human ductular hepatocytes. *Lab*. *Invest*. 83, 1395–1402. doi: 10.1097/01.lab.0000092229.93203.57

Huang, X., Ye, Q., Chen, M., Li, A., Mi, W., Fang, Y., et al. (2019). Zaytseva YY, O'Connor KL, Vander Kooi CW, Liu S, She QB. N-glycosylation-defective splice variants of neuropilin-1 promote metastasis by activating endosomal signals. *Nat*. *Commun*. 10, 3708. doi: 10.1038/s41467-019-11580-4

Jones, C. W., Gray, S. A.O., Theall, K. P., and Drury, S. S. (2018). Polymorphic variation in the *SLC5A7* gene influences infant autonomic reactivity and self-regulation: A neurobiological model for ANS stress responsivity and infant temperament. *Psychoneuroendocrinology* 97, 28–36. doi: 10.1016/j.psyneuen.2018.06.019

Kalli, A. C., and Reithmeier, R. A. F. (2018). Interaction of the human erythrocyte Band 3 anion exchanger 1 (*AE1*, *SLC4A1*) with lipids and glycophorin A: Molecular organization of the Wright (Wr) blood group antigen. *PLoS Comput*. *Biol*. 14, e1006284. doi: 10.1371/journal.pcbi.1006284

Kano, O., Tanaka, K., Kanno, T., Iwasaki, Y., and Ikeda, J. E. (2018). Neuronal apoptosis inhibitory protein is implicated in amyotrophic lateral sclerosis symptoms. *Sci*. *Rep*. 8, 6. doi: 10.1038/s41598-017-18627-w

Kapuria, V., Röhrig, U. F., Waridel, P., Lammers, F., Borodkin, V. S., van Aalten, D. M. F., et al. (2018). The conserved threonine-rich region of the *HCF-1PRO* repeat activates promiscuous OGT:UDP-GlcNAc glycosylation and proteolysis activities. *J*. *Biol*. *Chem*. 293, 17754–17768. doi: 10.1074/jbc.RA118.004185

Katoh, M., and Katoh, M. (2003). Identification and characterization of human Inscuteable gene in silico. *Int*. *J*. *Mol*. *Med*. 11, 111–116. doi: 10.3892/ijmm.11.1.111

Le, M. T., Lanaspa, M. A., Cicerchi, C. M., Rana, J., Scholten, J. D., Hunter, B. L., et al., (2016). Bioactivity-Guided Identification of Botanical Inhibitors of Ketohexokinase. *PLoS One*. 11, e0157458. doi: 10.1371/journal.pone.0157458

Leung, K. K., Suen, P. M., Lau, T. K., Ko, W. H., Yao, K. M., Leung, P. S. (2009). PDZ-domain containing-2 (*PDZD2*) drives the maturity of human fetal pancreatic progenitor-derived islet-like cell clusters with functional responsiveness against membrane depolarization. *Stem Cells Dev*. 18,979–990. doi: 10.1089/scd.2008.0325

Liao, M., Shi, J., Huang, L., Gao, Y., Tan, A., Wu, C., et al. (2014). Genome-wide association study identifies variants in *PMS1* associated with serum ferritin in a Chinese population. *PLoS One*. 9, e105844. doi:10.1371/journal.pone.0105844

Liu, J., Wang, Q., Zhang, R., Zhang, C., Lin, J., and Huang, X. (2018). Identification of *LINC01279* as a cell cycle-associated long non-coding RNA in endometriosis with GBA analysis. *Mol*. *Med*. *Rep*. 18, 3850–3858. doi:10.3892/mmr.2018.9387

Nagase, T., Ishikawa, K., Suyama, M., Kikuno, R., Miyajima, N., Tanaka, A., et al. (1998). Prediction of the coding sequences of unidentified human genes. XI. The complete sequences of 100 new cDNA clones from brain which code for large proteins in vitro. *DNA Res*. 5, 277–286. doi: 10.1093/dnares/5.5.277

Nakao, M., Shichijo, S., Imaizumi, T., Inoue, Y., Matsunaga, K., Yamada, A., et al. (2000). Identification of a gene coding for a new squamous cell carcinoma antigen recognized by the CTL. *J*. *Immunol*. 164, 2565–2574. doi: 10.4049/jimmunol.164.5.2565

Newman, J. A., Aitkenhead, H., Savitsky, P., and Gileadi, O. (2017). Insights into the RecQ helicase mechanism revealed by the structure of the helicase domain of human *RECQL5*. *Nucleic Acids Res*. 45, 4231–4243. doi: 10.1093/nar/gkw1362

Nguyen, P., Calderon, R., Rodriguez-Ledezma, Y., Araujo, K., and Bhandari, D. (2019). GIV/Girdin promotes cell survival during endoplasmic reticulum stress. *Mol*. *Cell Biochem*. 453, 79–88. doi:10.1007/s11010-018-3433-6

Perrault, I., Hamdan, F. F., Rio, M,. Capo-Chichi, J. M., Boddaert, N., Décarie, J. C., et al. (2014). Mutations in *DOCK7* in individuals with epileptic encephalopathy and cortical blindness. *Am*. *J*. *Hum*. *Genet*. 94, 891–897. doi:10.1016/j.ajhg.2014.04.012

Petrovics, G., Li, H., Stümpel, T., Tan, S. H., Young, D., Katta, S., et al. (2015). A novel genomic alteration of *LSAMP* associates with aggressive prostate cancer in African American men. *EBio*. *Medicine*. 2, 1957–1964. doi: 10.1016/j.ebiom.2015.10.028

Puchalapalli, M., Mu, L., Edwards, C., Kaplan-Singer, B., Eni, P., Belani, K., et al. (2019). The *Laminin-α1 Chain-Derived Peptide*, *AG73*, binds to syndecans on *MDA-231* breast cancer cells and alters filopodium formation. *Anal*. *Cell Pathol*. 2019, 9192516. doi: 10.1155/2019/9192516

Salichs, E., Ledda, A., Mularoni, L., Albà, M. M., and De la Luna, S. (2009). Genome-wide analysis of histidine repeats reveals their role in the localization of human proteins to the nuclear speckles compartment. *PLoS Genet*. 5, e1000397. doi: 10.1371/journal.pgen.1000397

Somekh, I., Marquardt, B., Liu, Y., Rohlfs, M., Hollizeck, S., Karakukcu, M., et al. (2018). Novel mutations in *RASGRP1* are associated with immunodeficiency, immune dysregulation, and *EBV*-induced lymphoma. *J*. *Clin*. *Immunol*. 38, 699–710. doi: 10.1007/s10875-018-0533-8

Tomkins, J. E., Dihanich, S., Beilina, A., Ferrari, R., Ilacqua, N., Cookson, M. R., et al. (2018). Comparative protein interaction network analysis identifies shared and distinct functions for the human *ROCO* proteins. *Proteomics* 18, e1700444. doi: 10.1002/pmic.201700444

Wallace, S. E., Regalado, E. S., Gong, L., Janda, A. L., Guo, D. C., Russo, C. F., et al. (2019). *MYLK* pathogenic variants aortic disease presentation, pregnancy risk, and characterization of pathogenic missense variants. *Genet*. *Med*. 21, 144–151. doi: 10.1038/s41436-018-0038-0

Wang, G., Shen, W., Liu, C. Y., Liu, Y., Wu, T., Cui, X., et al. (2017). Phosphorylase kinase β affects colorectal cancer cell growth and represents a novel prognostic biomarker. *J*. *Cancer Res*. *Clin*. *Oncol*. 143, 971–980. doi: 10.1007/s00432-017-2362-1

Wolosz, D., Walczak, A., Szparecki, G., Dwojak, M., Winiarska, M., Wolinska, E., et al. (2019). Deleted in Liver Cancer 2 *(DLC2*) protein expression in hepatocellular carcinoma. *Eur*. *J*. *Histochem*. 63, 1. doi: 10.4081/ejh.2019.2981

Yamanaka, R., Tabata, S., Shindo, Y., Hotta, K., Suzuki, K., Soga, T., et al. (2016). Mitochondrial Mg (2+) homeostasis decides cellular energy metabolism and vulnerability to stress. *Sci*. *Rep*. 6, 30027. doi: 10.1038/srep30027

Ye, L., Xiang, T., Zhu, J., Li, D., Shao, Q., Peng, W., et al. (2018). Interferon consensus *Sequence-Binding Protein 8*, a tumor suppressor, suppresses tumor growth andiInvasion of non-small cell lung cancer by interacting with the *Wnt/β*-catenin pathway. *Cell Physiol. Biochem*. 51, 961–978. doi: 10.1159/000495399

Zhang, H., Qiao, A., Yang, L., Van Eps, N., Frederiksen, K. S., Yang, D., et al. (2018). Structure of the glucagon receptor in complex with a glucagon analogue. *Nature* 553, 106–110. doi: 10.1038/nature25153

Zhang, W., Chen, Y., Swift, M. R., Tassi, E., Stylianou, D. C., Gibby, K. A., et al. (2008). Effect of *FGF-binding protein 3* on vascular permeability. *J. Biol. Chem*. 283, 28329–28337. doi: 10.1074/jbc.M802144200

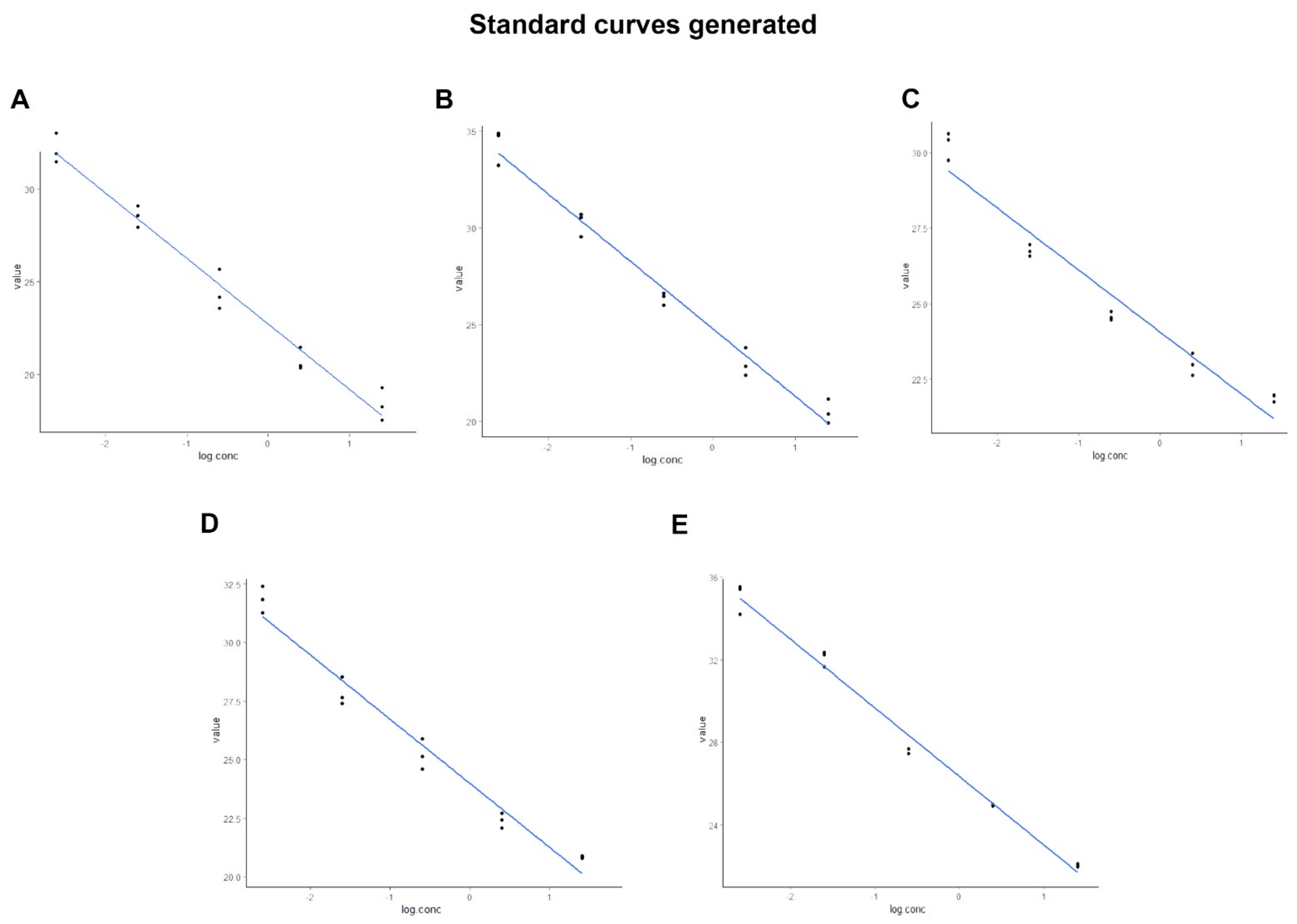

Supplementary Figure S1 | Standard curves generated for (A) *TMEM132D*, (B) *CCDC92*, (C) *ATP2A2*, (D) *PEBP1*, and (E) *GAPDH*. Linear dynamic range from 25 to 0.00025 ng/µl genomic DNA concentration extracted from green iguana (*Iguana iguana*) ($n = 10$). Regression lines were calculated as $R^2 = 0.9446–0.9879$, $p < 0.001$ for *TMEM132D*, *CCDC92*, *ATP2A2*, *PEBP1*, and *GAPDH*.

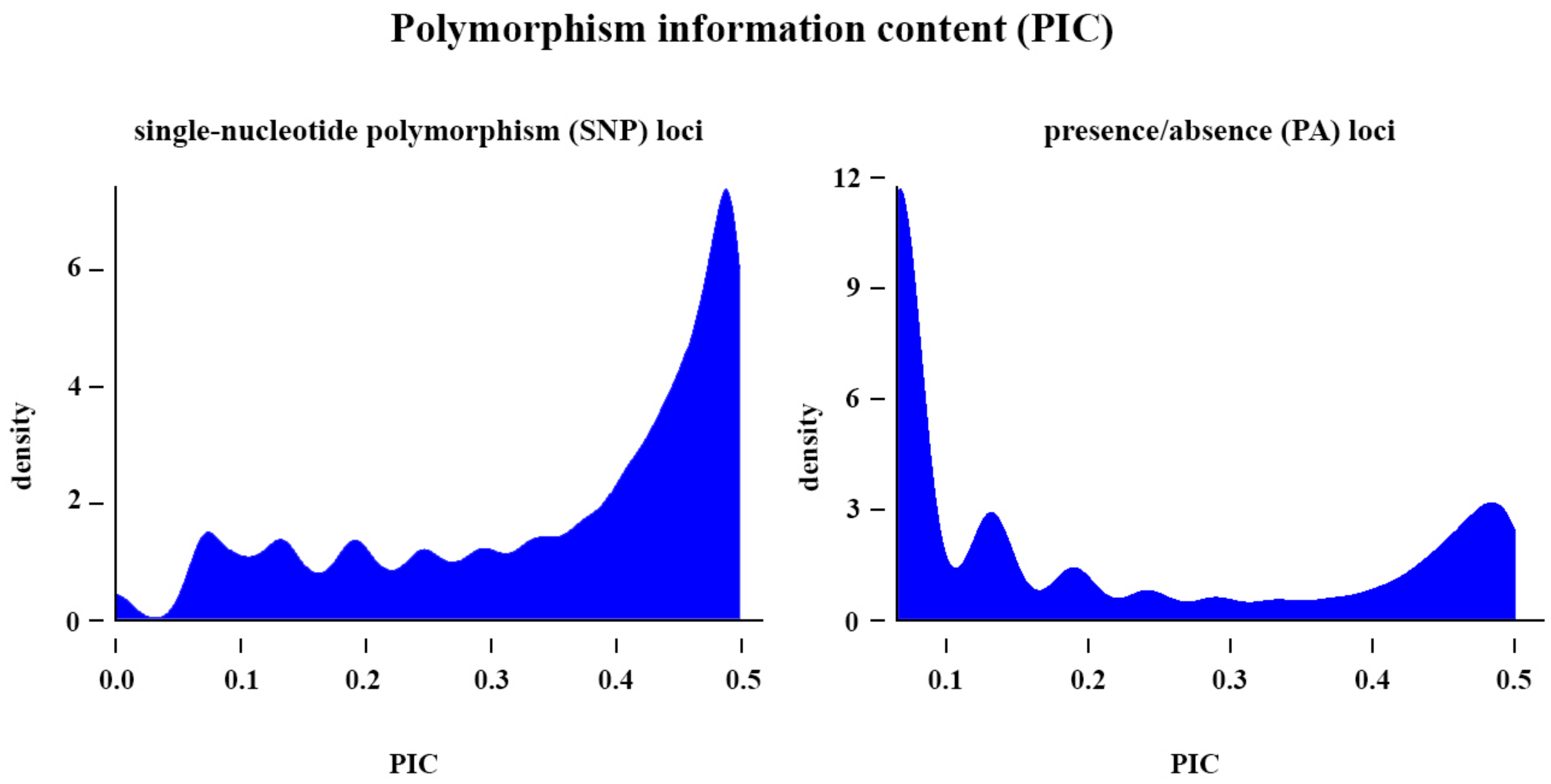

Supplementary Figure S2 | Density PIC plot for (A) single-nucleotide polymorphism (SNP) and (B) restriction fragment presence/absence (PA) loci.

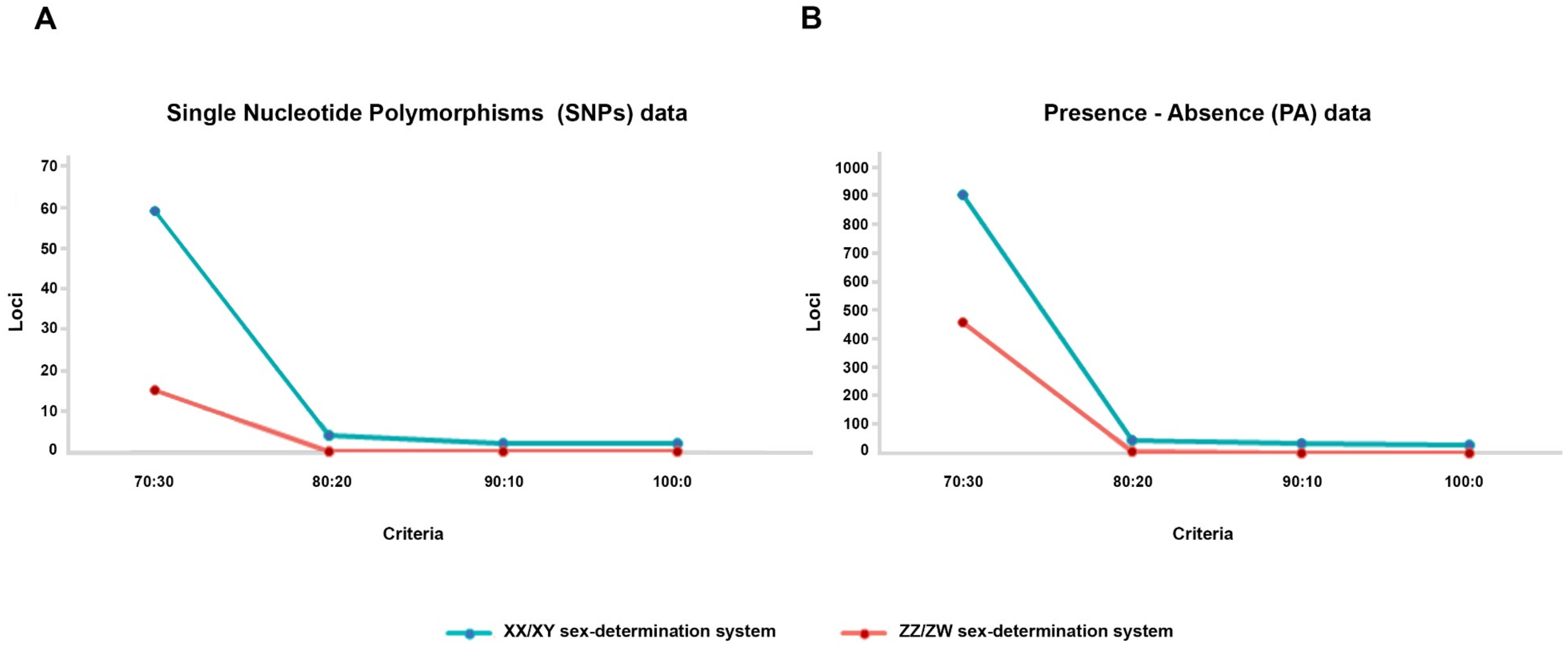

Supplementary Figure S3 | Number of loci in different hypotheses of sex-determination systems after filtering using different male:female ratios as criteria. The *x*-axis indicates the number of loci and the *y*-axis indicates the filtering criteria. (A) Restriction fragment presence/absence (PA) loci and (B) single-nucleotide polymorphism loci.

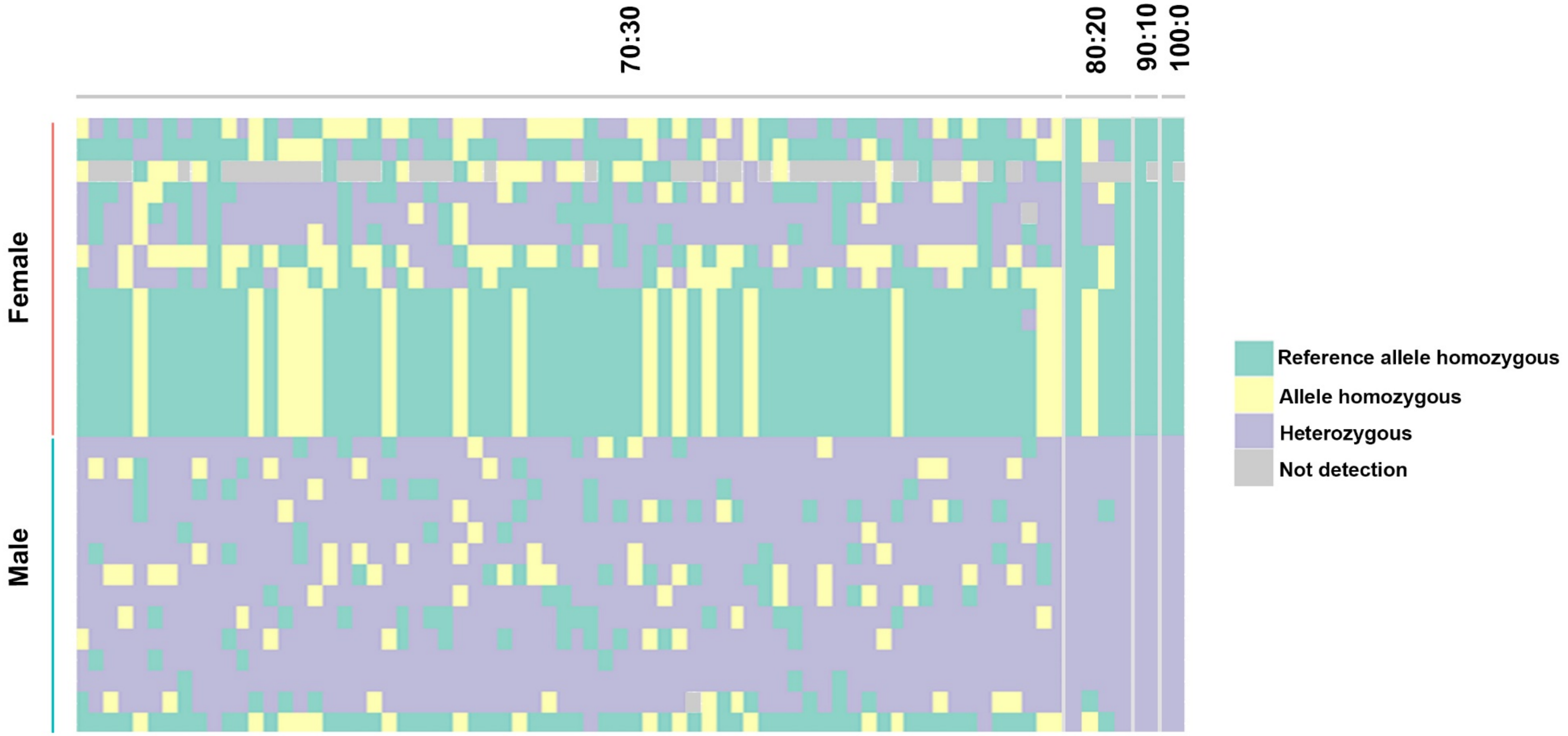

Supplementary Figure S4 | An index of the two perfectly sex-aligned sequences and the additional 59 sex-related sequences created with the ‘glPlot’ function implemented in the “dartR” R package. Blue indicates homozygosity to the reference allele, purple is indicative of heterozygosity, and red indicates homozygosity to the alternate SNP-containing allele. Sequences two (right of the figure) show perfectly sex-linked sequences; females are indicated in blue and males are indicated in purple.

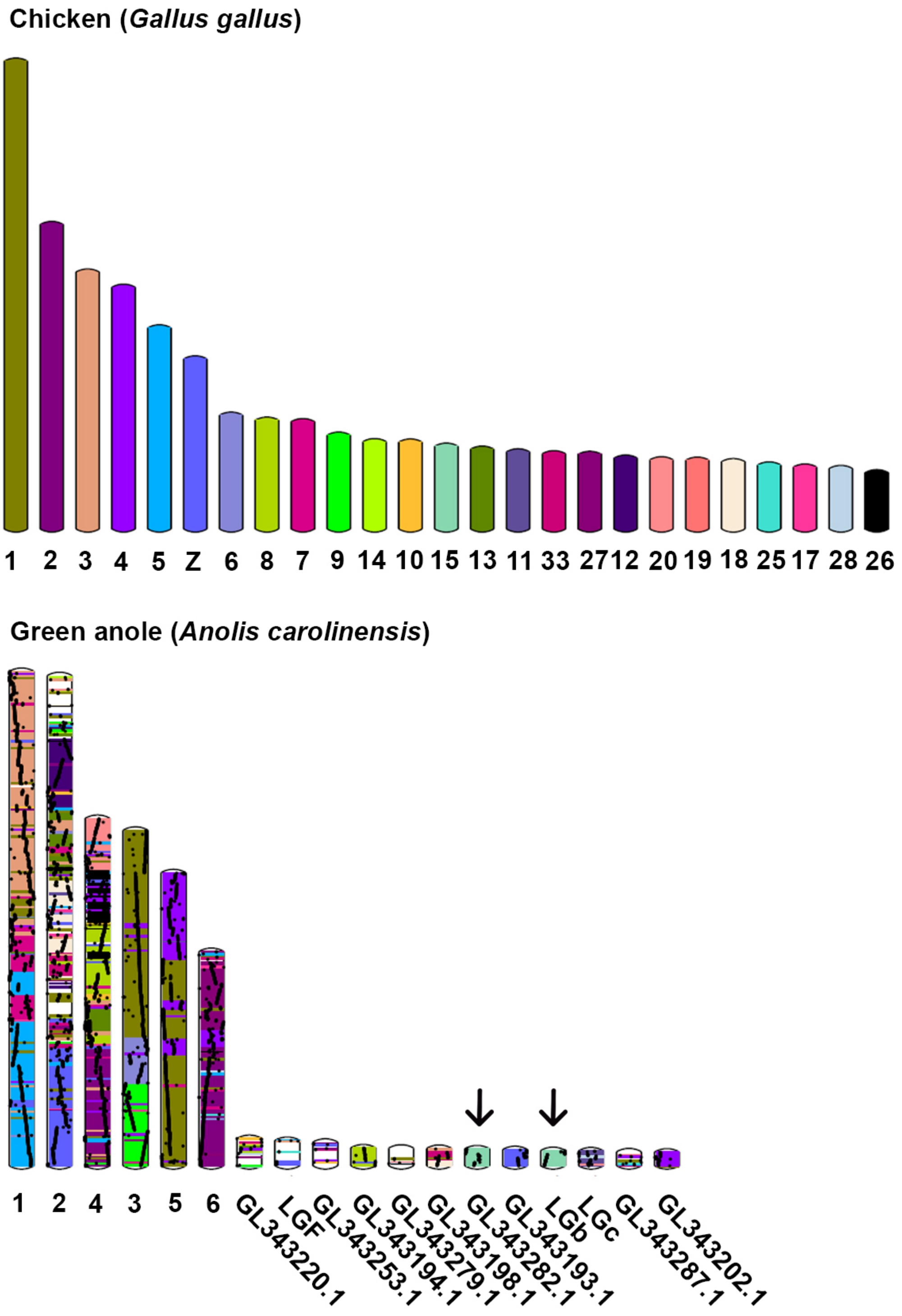

Supplementary Figure S5 | Segmental homology of green anole (*Anolis carolinensis*) and chicken (*Gallus gallus*). Segmental linkage homologies are derived from the Genomicus database (Louis *et al.*, 2014).
